# MAE-seq refines regulatory elements across the genome

**DOI:** 10.1093/nar/gkad1129

**Published:** 2023-12-01

**Authors:** Xiusheng Zhu, Qitong Huang, Lei Huang, Jing Luo, Qing Li, Dashuai Kong, Biao Deng, Yi Gu, Xueyan Wang, Chenying Li, Siyuan Kong, Yubo Zhang

**Affiliations:** Shenzhen Branch, Guangdong Laboratory of Lingnan Modern Agriculture, Key Laboratory of Livestock and Poultry Multi-omics of MARA, Agricultural Genomics Institute at Shenzhen, Chinese Academy of Agricultural Sciences, Shenzhen, 518120, China; Shenzhen Branch, Guangdong Laboratory of Lingnan Modern Agriculture, Key Laboratory of Livestock and Poultry Multi-omics of MARA, Agricultural Genomics Institute at Shenzhen, Chinese Academy of Agricultural Sciences, Shenzhen, 518120, China; Department of animal sciences, Wageningen University & Research, Wageningen, 6708PB, Netherlands; Shenzhen Branch, Guangdong Laboratory of Lingnan Modern Agriculture, Key Laboratory of Livestock and Poultry Multi-omics of MARA, Agricultural Genomics Institute at Shenzhen, Chinese Academy of Agricultural Sciences, Shenzhen, 518120, China; Shenzhen Branch, Guangdong Laboratory of Lingnan Modern Agriculture, Key Laboratory of Livestock and Poultry Multi-omics of MARA, Agricultural Genomics Institute at Shenzhen, Chinese Academy of Agricultural Sciences, Shenzhen, 518120, China; Shenzhen Branch, Guangdong Laboratory of Lingnan Modern Agriculture, Key Laboratory of Livestock and Poultry Multi-omics of MARA, Agricultural Genomics Institute at Shenzhen, Chinese Academy of Agricultural Sciences, Shenzhen, 518120, China; Shenzhen Branch, Guangdong Laboratory of Lingnan Modern Agriculture, Key Laboratory of Livestock and Poultry Multi-omics of MARA, Agricultural Genomics Institute at Shenzhen, Chinese Academy of Agricultural Sciences, Shenzhen, 518120, China; Shenzhen Branch, Guangdong Laboratory of Lingnan Modern Agriculture, Key Laboratory of Livestock and Poultry Multi-omics of MARA, Agricultural Genomics Institute at Shenzhen, Chinese Academy of Agricultural Sciences, Shenzhen, 518120, China; Shenzhen Branch, Guangdong Laboratory of Lingnan Modern Agriculture, Key Laboratory of Livestock and Poultry Multi-omics of MARA, Agricultural Genomics Institute at Shenzhen, Chinese Academy of Agricultural Sciences, Shenzhen, 518120, China; Shenzhen Branch, Guangdong Laboratory of Lingnan Modern Agriculture, Key Laboratory of Livestock and Poultry Multi-omics of MARA, Agricultural Genomics Institute at Shenzhen, Chinese Academy of Agricultural Sciences, Shenzhen, 518120, China; Shenzhen Branch, Guangdong Laboratory of Lingnan Modern Agriculture, Key Laboratory of Livestock and Poultry Multi-omics of MARA, Agricultural Genomics Institute at Shenzhen, Chinese Academy of Agricultural Sciences, Shenzhen, 518120, China; Shenzhen Branch, Guangdong Laboratory of Lingnan Modern Agriculture, Key Laboratory of Livestock and Poultry Multi-omics of MARA, Agricultural Genomics Institute at Shenzhen, Chinese Academy of Agricultural Sciences, Shenzhen, 518120, China; Shenzhen Branch, Guangdong Laboratory of Lingnan Modern Agriculture, Key Laboratory of Livestock and Poultry Multi-omics of MARA, Agricultural Genomics Institute at Shenzhen, Chinese Academy of Agricultural Sciences, Shenzhen, 518120, China; Kunpeng Institute of Modern Agriculture at Foshan, Foshan, 528225, China

## Abstract

Proper cell fate determination relies on precise spatial and temporal genome-wide cooperation between regulatory elements (REs) and their targeted genes. However, the lengths of REs defined using different methods vary, which indicates that there is sequence redundancy and that the context of the genome may be unintelligible. We developed a method called MAE-seq (Massive Active Enhancers by Sequencing) to experimentally identify functional REs at a 25-bp scale. In this study, MAE-seq was used to identify 626879, 541617 and 554826 25-bp enhancers in mouse embryonic stem cells (mESCs), C2C12 and HEK 293T, respectively. Using ∼1.6 trillion 25 bp DNA fragments and screening 12 billion cells, we identified 626879 as active enhancers in mESCs as an example. Comparative analysis revealed that most of the histone modification datasets were annotated by MAE-Seq loci. Furthermore, 33.85% (212195) of the identified enhancers were identified as de novo ones with no epigenetic modification. Intriguingly, distinct chromatin states dictate the requirement for dissimilar cofactors in governing novel and known enhancers. Validation results show that these 25-bp sequences could act as a functional unit, which shows identical or similar expression patterns as the previously defined larger elements, Enhanced resolution facilitated the identification of numerous cell-specific enhancers and their accurate annotation as super enhancers. Moreover, we characterized novel elements capable of augmenting gene activity. By integrating with high-resolution Hi-C data, over 55.64% of novel elements may have a distal association with different targeted genes. For example, we found that the Cdh1 gene interacts with one novel and two known REs in mESCs. The biological effects of these interactions were investigated using CRISPR-Cas9, revealing their role in coordinating Cdh1 gene expression and mESC proliferation. Our study presents an experimental approach to refine the REs at 25-bp resolution, advancing the precision of genome annotation and unveiling the underlying genome context. This novel approach not only advances our understanding of gene regulation but also opens avenues for comprehensive exploration of the genomic landscape.

## Graphical Abstract

**Graphical Abstract:**
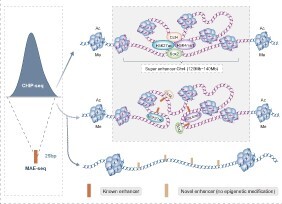

## Introduction

Transcriptional regulatory elements (REs) are nucleotide sequences of genes involved in genetic transcription regulation. Accurate identification of REs is fundamental for annotating genomes and understanding gene expression patterns (1). According to the ENCODE (Encyclopedia of DNA Elements) project, 74.7% of the genome is transcribed, with 56.1% associated with modified histones, 15.2% in open-chromatin areas, 8.5% binding transcription factors (TFs), and 4.6% comprising methylated CpG dinucleotides (2). However, mounting evidences suggest that many of these transcribed regions are nonfunctional (3–5). Whitfield *et al.* found that less than 50% of predicted transcription factor binding sites (TFBSs) from ENCODE made a functional contribution to the promoter activity in four cell lines (K562, HCT116, HT1080 and HepG2) (6). Non-coding loci without epigenomic signals can still be functional in the human genome (7). Despite advances in sequencing technology, genome annotation methods remain largely unchanged, leading to prevalent annotation errors (8). Therefore, there is a growing need for precise RE identification.

REs control gene expression temporally and spatially, playing pivotal roles in biological development, differentiation, and disease occurrence (9). The most common experimental method for genome-wide identification of transcriptional REs is chromatin immunoprecipitation, followed by high-throughput sequencing (ChIP-Seq) (10) and chromatin-based methods. Unfortunately, due to the limitations of nucleosome structure or technical means, the lengths of the REs identified vary a lot. In eukaryotes, 147 bp of DNA are wrapped around an octamer of histones (two copies of each H2A, H2B, H3, and H4) to form the smallest unit of chromatin, the nucleosome (11). Therefore, it is tough for existing chromatin-based methods to refine the REs span to less than ∼147 bp. For example, the RE length revealed by DHS-seq, FAIRE-seq and ChIP-seq is ∼200–500 bp, ∼200–1000 bp and ∼200–700 bp, respectively (12–14). As a non-chromatin-based method, the length of the recently developed STARR-seq is 500–850 bp (15,16). These differences in length suggest that the sequences of annotated REs likely have redundancy. Meanwhile, the RE size predicted in the ENCODE project is much larger than its actual size (17). This implies that the information hidden in a large part of these discriminated nucleotides has not been properly interpreted, and that their potential functions might remain unknown. It indicates that accurate identification and characterization of REs need to be further clarified and their control transcription in a cell-type specific manner might enlighten novel molecular mechanisms of gene regulation and diseases (18).

Massively parallel reporter assays (MPRAs) have been widely used for screening REs, including episomal and self-transcribed active regulatory region sequencing (STARR-seq) (19). hey sequence mRNA abundance in transfected cells to identify enhancer sequences. However, constructing output libraries for MPRAs is expensive and time-consuming. Additionally, bioinformatic methods have identified thousands of short core motifs spanning 6–14 nucleotides, but these motifs do not function as individual REs (20).

Cell type-specific enhancers, crucial in dictating cellular identity, frequently engage cell-specific transcription factors, including super enhancers (21,22). Existing enhancer investigations often involve multiple transcription factors, leading to complex analyses due to the variability in sequence lengths. Despite their large span, functional regions within super enhancers have been shown to be only modestly sized (23), necessitating experimental efforts for comprehensive annotation. Enhancer functionality hinges on cofactors, such as the COFs family (e.g. CDK9, BRD4) and chromatin-remodeling complexes (CRCs) like SMARCC1 and SMARCB1 (24–26). The interplay between enhancers, cofactors, and the epigenetic landscape merits further exploration.

In this study, we combined the concept of motifs with MPRAs to design short random nucleotides libraries with different lengths for high throughput discovery of functional ERs with minimum length, termed MAE-seq (Massively Active Enhancer for Sequencing). This approach allows high-throughput discovery of functional enhancer REs with minimum length. To screen functional enhancers, we used a minimal promoter to test the activity of the upstream enhancer of the fluorescent reporter gene, which remains silent in the absence of an enhancer or basal expression. Our results determined that ∼25 bp is a suitable length for functional enhancer screening. We applied MAE-Seq in both human and mouse cells, identifying tens of thousands of 25-bp enhancer REs in these cell types. These data refine the sequence context of REs and uncover precise genetic information throughout the genome.

## Materials and methods

### Screening vector

The reporter vector, pMX-mP-mCherry based on the modified pMX-GFP retroviral vector (Cell Biolabs, USA), was used in the study. Minimal promoter and mCherry (miniP-mCherry) fragments were obtained from a modified pGL4.23 luciferase reporter vector (Promega, USA). Briefly, PGL4.23 was digested with XbaI/NcoI restriction enzymes to remove the original coding sequences of the luc2 reporter gene and replace it with mCherry. The miniP-mCherry fragment was then amplified with primers containing NcoI or NotI restriction sites. Synthetic DNA fragments were cloned into NcoI and NotI digested pMX-GFP vectors. The resulting screening vector (see supplement file), pmx-mchery, was double digested with Hind III and xhoI to form a linearized vector that can be used for library generation.

### Generation of random DNA libraries

A random DNA library was constructed following a two-step process (DNA fragment synthesis and seamless assembly cloning) (Supplementary Figure S1C). Random DNA fragments comprising different lengths of DNA fragments, homologous arms and Illumina adapters at both ends (CTAACTGGCCGGTACCTGAGCTCGCTAGCCTCGAGTCGTCGGCAGCGTCAGATGTGTATAAGAGACAG-Randomsequence-CTGTCTCTTATACACATCTCCGAGCCCACGAGACAAGCTTAGACACTAGAGGGTATATAATGGAAGCTCG) were synthesized by GENEWIZ (Suzhou, China) and inserted into a reporter vector through homologous recombination methods following the Clone Express II One Step Cloning Kit protocol (Vazyme; Cat. No. C112). Sanger sequencing was conducted to check the quality of the library clones. Combine 80 homologous recombination reaction systems and purify them with 1.8 times AMPure XP Beads, transform the purified product into electrocompetent cells (ThermoFisher Scientific; Cat. No. C6400), and transfer the transformed competent cells to 10L LB medium Amplify. When the OD value of the bacterial solution is 1.0, extract the plasmid according to the instructions of the kit (QIAGEN; Cat. No. 12362). These plasmid libraries were transfected into cells, and then cell genomic DNA was extracted. Perform PCR amplification with 100ng genomic DNA as template (95°C 45s; followed by 15 cycles of 98°C for 15 s, 60°C for 30 s, 72°C for 3 min), and combine 20 PCR reactions. The product was recovered by 2% agarose gel electrophoresis for 50 min (120v, 130mA) and sent for high-throughput sequencing, the data from the sequencing machine is the input library.

**Figure 1.**
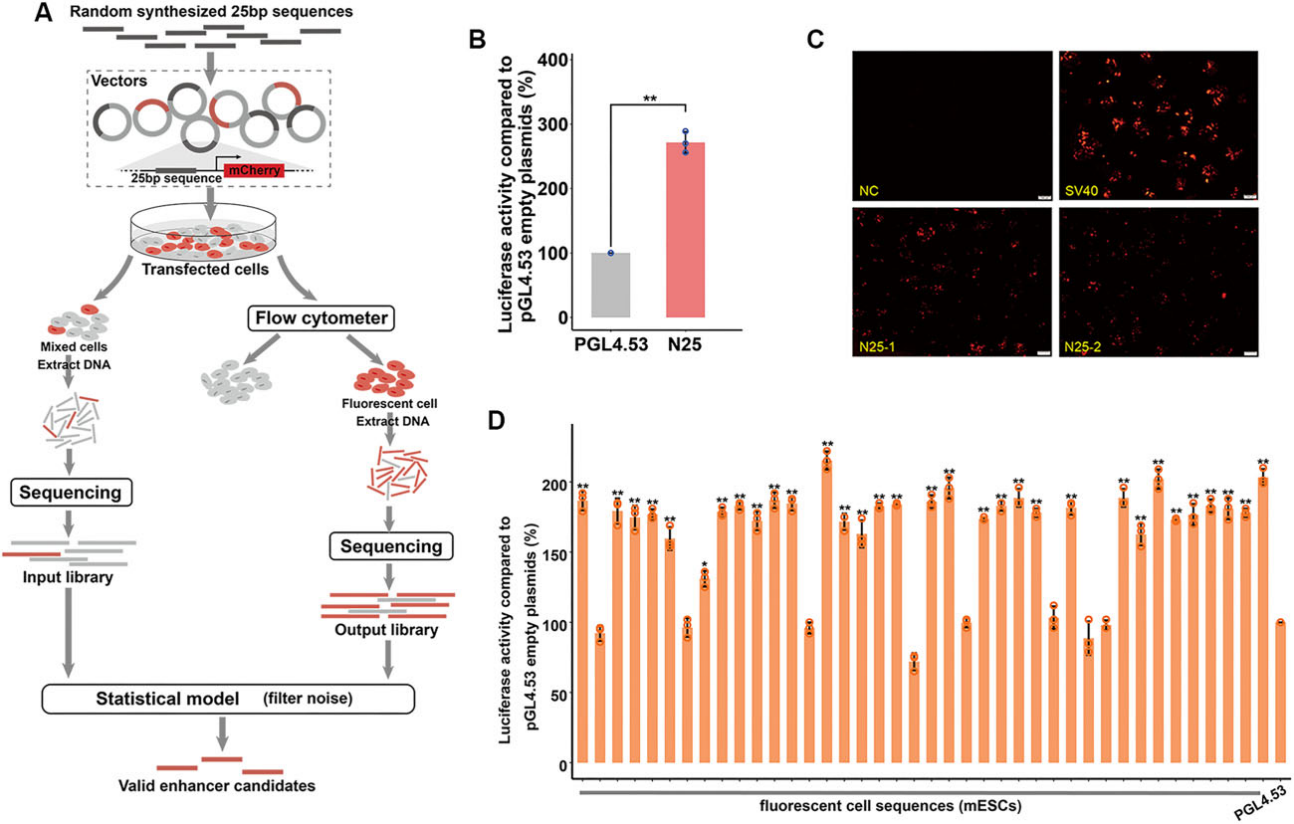
MAE-seq development. (**A**) The fragments to be screened were constructed into mCherry fluorescent reporter vector with mini promoter to form a plasmid library, and the cells after transfection of the plasmid library were divided into two groups, one group extracted all cell DNA specific amplification without fluorescence sorting and then high throughput sequencing (input), and one group extracted fluorescent cells by flow screening and then high throughput sequencing (output). The final enhancers are determined by joint analysis of input and output using the model we developed. (**B**)The dual luciferase activity of 25 bp DNA sequence was verified. The 25 bp sequence is selected from the reported long sequence enhancer. We selected 25 bp sequences of many different fragments, and only 25 bp with enhancer activity is displayed here to prove that the 25 bp DNA sequence has enhancer function. empty pGL4.53 plasmid was used as the control for baseline luciferase activity. The y axis represents the percentage of luciferase activity compared to pGL4.53 empty plasmids in the respective cells (*n* = 3 biological independent samples; bars show mean value ± s.e.m.; ns: non-significant, ** *P*< 0.01, calculated using *t*-test). (**C**) Intracellular activity validation of MAE-seq data. NC, empty reporter vector; SV40, SV40 enhancer; N25-1, N25-2, two randomly selected sequences of MAE-seq data. Constructed reporter vectors are transfected into mESCs to validate enhancer activity. NC is negative control and SV40 is a positive control. (**D**) The luciferase reporter system was used for activity validation of nine randomly selected MAE-seq loci in mESCs. empty pGL4.53 plasmid was used as the control for baseline luciferase activity. The y axis represents the percentage of luciferase activity compared to pGL4.53 empty plasmids in the respective cells (*n* = 3 biological independent samples; bars show mean value ± s.e.m.; ns: non-significant, ** *P*< 0.01, calculated using *t*-test).

### Cell culture and transfection

293T cell cultures were inoculated into 100 mM DMEM (ThermoFisher; Cat. No. 10566024) containing 10% FBS (Gibco; Cat. No.10437028) and 1% Penicillin Streptomycin P/S (Sigma; Cat. No. P0781) at a 37°C cell incubator. Transfection of plasmid libraries (1 ng DNA/1.2 × 10^7^ cells) was performed using cells at 70–80% confluence using HD Transfection Reagent (FuGENE, Cat. No. E2311). Fluorescence cells were collected 48 hours after transfection.

E14 ESCs cultures were inoculated into a culture solution containing 100 mM ES medium (KO-DMEM (Gibco, 10829-018), 15%FBS (Gibco; Cat. No. 10099), 0.2 mM GlutaMAX™ Supplement (Gibco; Cat. No. 35050061), 0.1 mM MEM nonessential amino acids (NEAA) (Gibco; Cat. No. 11140–122), 50 U/ml penicillin/50 μg/mL streptomycin (Gibco; Cat. No. 15140-122), 1 U/ml LIF (Chemicon; Cat. No. ESG-1106), 0.1 mM 2-mercaptoethanol (BME) (Gibco; Cat. No. 21985023), 0.16 mM Vitamin C (Sigma; Cat. No. A4034), 1 μM PD0325901 and 3 μM CHIR-99021 (CT99021). Transfection of plasmid libraries (1μg DNA/1 × 10^6^cells) was performed with cells at 70–80% confluence using Lipofectamine 3000 Transfection Reagent (Invitrogen, Cat. No. L3000075). Fluorescence cells were collected 48 h after transfection.

### Generation of output libraries(one-step)

DNA was extracted(Vazyme; Cat. DC112) from the collected fluorescent cells. Components were added according to the TruePrep DNA Library Prep kit (Vazyme; Cat.TD202) and followed by one-step PCR (72°C for 3min, 98°C for 30s; followed by 5–15 cycles of 98°C for 15 s, 60°C for 30 s, 72°C for 3 min; 72°C for 5 min). The product was recovered by 2% agarose gel electrophoresis for 35 min (120v, 130mA) and sent for rubber recycling. The data obtained by high-throughput sequencing of the gum recovery products were output libraries. Sequence after one-step PCR amplification is 5′-AATGATACGGCGACCACCGAGATCTACACIIIIIIIITCGTCGGCAGCGTCAGATGTGTATAAGAGACAG-NNNNNN-CTGTCTCTTATACACATCTCCGAGCCCACGAGACIIIIIIIIATCTCGTATGCCGTCTTCTGCTTG-3′, IIIIIIII: Index 2 (i5), IIIIIIII: Index 1 (i7), -NNNNNN-: random sequence.

### Luciferase assay

Synthesize sequences that require validation(GENEWIZ, China). These fragments were then inserted in front of the PGK promoter of the luciferase plasmid pGL4.53 (Promega) using Gibson assembly. Cells were then co-transfected with the pRL-CMV Renilla reporter vector and the pGL4.53 vector with the silencer sequence inserted. The luciferase assay was performed using the Dual-Glo Luciferase Assay Kit from Promega according to the manufacturer's protocol. The original luciferase plasmid without any insertion was used as the control. All the luciferase assays were from three independent transfections performed on different days.

### ‘LER’ experimental assay

Five 25-bp loci of MAE-seq enhancers that overlapped peaks of ChIP-seq data were randomly selected for analysis. The activities of enhancers together with their 5′ and 3′ 200bp-flanking regions were validated, respectively. We termed MAE-seq enhancer, 5′ and 3′ flank sequences as ‘E’, ‘L’ and ‘R’ respectively. Six test groups were set up as follows: L, L-E, E, E-R, R, LR and L-E-R. These synthesized fragments (Sangon Biotech) were ligated into the PGL4.53 vector using the homologous recombination method. Their activity was detected by a luciferase reporter system.

### Endogenous activation of enhancers and gene expression assay

The U6-BpiI-GFP vector was digested with BbSI, and the target fragment was recovered using a gel recovery kit (D4008, ZYMO). The first base of the gRNA sequence design starts with G, and the top three are selected for use. The rules for adding cohesive end bases in gRNA synthesis are as follows. F: CACC-gRNA, R: AAAC-gRNA. where gRNA represents the sequence given by the software during design. Dissolve gRNA primer with ddH2O at a concentration of 100uM, each take 1 μl, 6.5 μl ddH_2_O, 1 μl 10 × 4 ligation buffer (NEB), 0.5 μl T4 PNK (NEB M0201S) to configure a 10 μl annealing reaction system, and perform annealing and phosphoric acid in a PCR machine change. Procedure: 37°C, 30 min, 95°C 5 min, gradient cooling to 25°C, 5°C/min. 1:200 dilution of the annealing product. Take 6μl annealing product, add 1 μl linearized plasmid, 1 μl 10 × 4 ligation buffer, 1 μl T4 DNA ligase, 1μl ddH_2_O, configure it into a 10 μl ligation reaction system, and ligate at room temperature for 4–6 h. Then proceed to transformation, AMP plate coating, and clone sequencing. Sequencing primer: hU6-F: gagggcctatttcccatgatt. The dSpCas9-VP64, U6-BpiI-gRNA-GFP were transfected into the cells at a ratio of 1:1, and RNA was collected at 24, 48 and 72 h to quantitatively detect the expression of target genes. If the transfection efficiency is low, the flow can be enriched. The control group was transfected with dSpCas9-VP64 and U6-BpiI-GFP empty vectors. The mRNA was reverse-transcribed into cDNA using the reverse transcription kit (HiScript III RT SuperMix for qPCR, R323-01, Vazyme). The qPCR kit (AQ101, TransGen) was used for real-time quantitative PCR to detect the changes of target genes.

### Establishment of an enhancer knockout cell

The ssDNA designing follows below principles: (i) the cleavage site of Cas9 protein in the genome is taken as the center; (ii) upstream and downstream sequences of 90bp length are intercepted as the left and right homologous arms of ssDNA respectively; (iii) enhancer is deleted in downstream sequence; (iv) the length of designed ssDNA is 180 bp; (v) the NGG of PAM sequence is mutated into NCG to prevent the repeated cleavage of Cas9; (vi) Hind III endonuclease site is designed to facilitate genotyping of knockout cells. Sequences of ssDNA related to Ce1 and Ce3 are presented as follows.

>ssDNA_Ce1

TTTAATGGCTGCTCTCTCTCTCTCTCTCTCTCTCTCTCTCTCTCTCTCTCTCTCTCTCTCTTTCAAAGCCACTTGGCCTCTTTATTAAGCTTCACGTGTCTTGAGACTTGATTCCTGTCTAGATCCCTCTTATTCCAGAAATGACAGGATTTCCATGTACCTGCATCAATGCATCTTAGC

>ssDNA_Ce2

CCCACCAGTCCTGGGGCTCTGGTATTTATACTTTCTCCAGAATCCCCAGAACTAAACCATCTGCAGCTGACAAAGATCATGCCCCGTCCTAAAGCTTTGAGACAGTCACAATTAACGGCTGTAGACAAACTGAAGCAGTCCCATATATATATTCATATAACATAACCGAAATCAAAATTC

>ssDNA_Ce3

GGAGTGTGGGTAATGAGTGACTGGATCAGTGTCGAAGGGAACAGTTTCCGCATCTTTACTTATGGAAGGGTCTTAAACAAGCTTACTGTGTAGCCGTTTGCACAGCCAGATTTAATTACTACTGCCTGTCTCCTCACGGGTGTTTAAAATAAACTTGCATATTCTGTTACAGTTTAAGAA

Then the enhancer knockout cells were genotyped. The genomic DNA of enhancer knockout cells was used as a PCR template. The primer sequences are as follows. Ce1_F: CTCAGACTCAGAGATCCCTC, Ce1_R: CTCTGCCTCTTGTAGGGAAG; Ce2_F: TCCATCAGATTGGCCTGTAG, Ce2_R: AACTGCTCTGAGCACCAGAC; Ce3_F: GTCAGTGCTCTTATCTGCTG, Ce3_R: GCAGAGCTGTCTACATAGTG.

The lengths of PCR products related to Ce1 to Ce3 are 888, 1029 and 1022 bp, respectively. They were digested by Hind III for genotyping detection. If the product is wild type, there is no Hind III restriction site in these sequences, which could not be cleaved by the enzyme. This was used as the criterion to determine the genotype of knockout cells.

### Cell counting kit-8 assay

Cell proliferation was determined using the Cell Counting Kit-8 (C0037, Beyotime). Briefly, 3 × 10^4^ cells were seeded in a well of a 24-well flat-bottomed plate and incubated for 12 h. Replace 1 ml of fresh medium and add 100μl CCK8, incubate for 2 h in the incubator. The absorbance at 450 nm was measured using a microplate reader (Bio-Rad, Hercules, CA, USA). The experiments were repeated at least three times.

### 5-Ethynyl-2-deoxyuridine (EdU) assay

About 1.6 × 10^5^ cells per well were inoculated in a 6-well plate, EDU (ST067, Beyotime) was added after 12 h of culture to make the final concentration 10uM and the cells were incubated for 2 h in an incubator. After removing the medium, 1ml fixative (4% Paraformaldehyde) was added, and cells were fixed at room temperature for 15 min. After removal of fixative, and 1ml permeabilization solution was added and cells were incubated at room temperature for 15 min. Later 0.5ml Click reaction solutions were added and cells were incubated for 30 min in the dark. Finally, cells were stained with Hoechst. Results of the EdU assay were observed and analyzed under an EVOS M5000 microscope (Thermo Fisher Scientific).

### Cas13d-mediated RNA interference

The Cas13d system was harnessed as a versatile tool for targeted gene interference. In a concise overview, the Cas13d plasmid was subjected to enzymatic digestion employing restriction endonuclease BbsI. Subsequently, primers designed to target the specific gene of interest were meticulously ligated onto the plasmid vector using the high-fidelity T4 DNA ligase. Following plasmid construction, cellular transfection was executed, and the ensuing impact on the expression of the target protein was assessed following a 48-hour interval.

### RNA-seq library construction

RNA was isolated using Trizol (Invitrogen) and Kit (Mei5Bio,Cat No.MF167) from mESCs. Three libraries (biological duplicates) were constructed by the VAHTSTM Total RNA-seq (H/M/R) Library Prep Kit (Vazyme Biotech, China). The library construction method was referred to in the instructions for the library prep kit. 0.5 μg total RNA was diluted with nuclease-free Water in a nuclease-free PCR tube. Then the rRNA probe (H/M/R) was hybridized with a temperature gradient descending method (95–22°C, 0.1°C/sec). rRNA and genome DNA was digested with RNase H and DNase I successively (37°C for 30 min). The ribosomal-depleted RNA was purified with VAHTS RNA Clean Beads (in 2.2×) in a magnetic stand. Then the RNA sample was eluted with Frag/Primer Buffer for fragmentation (For 150–200 bp insert: incubate at 94°C for 8 min). Double Strand cDNA was synthesized with 1st and 2nd Strand Enzymes and purified with VAHTS DNA Clean Beads (in 1.8x). After dA-tailing and RNA Adapter Ligation, the sample was purified, and size was selected with two steps of DNA Clean Beads (in 1x) incubation. PCR Primer Mix and Amplification Mix were used for library amplification. The quality of purified PCR product was determined by using an Agilent Technologies 2100 Bioanalyzer. Multiplexed massively parallel sequencing (paired ends of 150 base pairs [bp]) was conducted using the Illumina platform.

### Mapping simulation

To figure out the optimal length of candidate DNA fragments to increase the library saturation and alignment accuracy, we used BEDTools (27) to randomly extract sequences with different lengths (20–60 bp, 1bp interval) from human reference genome and align them to hg19 by bowtie (version 1.2.2) (28), bowtie2 (version 2.1.0) and bwa (version 0.7.5a-r405) (29), respectively. The unique mapping rate was counted for further analysis. In this study, each group had three repetitions, 1 million sequences with a specific length in each repetition.

### Alignment of input and output libraries

MAE-seq data have been uploaded to the GEO database (GSE149028). The input library and output libraries raw data (raw reads) of fastq format were firstly trimmed by cutadapt (v1.16) (30) to remove adapter. Then, the remaining reads were aligned to the mm9/hg19 reference genome using bwa (version 0.7.5a-r405). Uniquely mapped reads with 25 bp inserted and no more than two mismatches from either bwa (29) were kept. The genomic start and end coordinates of these reads were then used for downstream processing.

### Normalization

The enrichments of input and output datasets are not uniform. The observation of fragments will be affected by library size and PCR cycles. Theoretically, the larger the library is, the greater the probability of observed frequency holds. Meanwhile, the 25 bp-fragments mapped to the genome are faultlessly aligned without transplacements. Due to this characteristic, the sequencing data cannot be removed from PCR duplicates, and the number of cycles will also influence the quantity. Therefore, we designed a quantitative function to normalize the fragment counts and compute the normalization coefficient:


\begin{eqnarray*}{\mathrm{f}}\left( x \right) = \frac{x}{{N \times \log {{\left( {1 + p} \right)}}^c}}\omega \end{eqnarray*}

The observation is at a given location where the *x* is reads observation in a given location. *N* is the total number of reads. *p* is the PCR amplification efficiency (default = 1). *c* is the number of PCR cycles. ω is the zoom factor.

It is known that the less value of parameter in Possion test will bring a lower significance, so we set the zoom factor as the compensation to fit the test.

The zoom factor ω is calculated by the following formula:


\begin{eqnarray*}\omega = 10\left( {\left[ {{\mathrm{log}}10\left( {{\mathrm{MIN}}\left( {x,y} \right)} \right)} \right] + 1} \right)\end{eqnarray*}

Where the MIN function is the minor value of the total input and output read counts. And x,y are the total read counts of input and output libraries, respectively.

### Significant difference analysis and positive signal (MAE-seq enhancer) selection

To achieve significant difference analysis between input and output data, we first determined the data distribution of MAE-seq data. Obviously, the probability of a random 25bp fragment that can be matched to a location in the reference genome is infinitesimal. Meanwhile, the number of repetitions is very large, and events are independent. Therefore, the MAE-seq data follows a Poisson distribution, and the probability function is:


\begin{eqnarray*}P\left( {x = k} \right) = \frac{{{\lambda }^k}}{{k!}}{e}^{ - \lambda }\end{eqnarray*}

\begin{eqnarray*}\lambda = n \times p\end{eqnarray*}

where *k* is represents the number of observations. *n* is the total number of reads. *P* is the probability of the observation.

Then, we applied the Poisson test to calculate the significant difference and used the Benjaminiand Hochberg test to select the loci with Q values that are lower than 0.05 as effective signals. To avoid incorrect filtering, we set a compensation value for the loci appearing in the output dataset but not in the input dataset. This part was processed by R(4.0.2).

The compensation value is also playing a key role in the Poisson test. The higher the value, the more rigorous the result, and vice versa. There are two factors that determine the validity of this compensation value: (i) the non-overlap rate between MAE-seq signals and annotated regions; (ii) the remaining valid signals. To determine the value, we set 0.1–3 times the input data normalization coefficient as the compensation value. When the compensation value is equal to 1 normalization coefficient, the result takes into account both these factors (Supplementary Figure S1G).

### Annotating enhancers in the genome

The coordinates of enhancers in the genome were annotated by HOMER (31). To annotate the enhancer location in terms of important genomic features, the genome was annotated in the following categories: TTS (by default defined from −100 bp to +1 kb), intergenic, exon, intron, 5′UTR and 3′UTR. Each enhancer was sorted into a different category if its locus fell within the sequence of a specific category.

### Endogenous chromatin environment of enhancer candidates

To study the endogenous chromatin environment of enhancer candidates, data from DNase-seq (ESC), ATAC-seq (293T), ChIP-seq for histones (H3K4me1 and H3K27ac of ESC and 293T) and transcription factors (Sox2, Nanog and Oct4 of ESC) were downloaded from the ENCODE or GEO database (Supplementary Table S2). Since most of the features, such as histone modifications, were found at nucleosomes near enhancer elements but not the exact location of the enhancer elements, we extended the region of peak by ±2500 bp. The proportion of overlap between the genome position of enhancers and the extended peaks was calculated by BEDTools (27). In addition, the enrichment of H3K4me1 around the MAE-seq enhancers was analyzed and shown by deepTools (32). The overlap between MAE-seq enhancers and H3K27ac, Nanog, Oct4, Sox2, DNase-seq were calculated by BEDTools and visualized by R.

### RNA-seq data analysis

First, the quality control was conducted on the RNA-seq data by using FatQC (v0.11.5) (33). Then, to ensure the quality of RNA-seq data used in further analysis, rRNA sequences were first filtered out by SortMeRNA v2.0(default parameter). RNA-seq data associated with this study are derived from the GEO database (GSE115750). Clean reads were obtained from the raw data by removing adapter-containing reads, poly-N-containing reads, and low-quality reads using Trimmomatic (version 0.36) (32). All downstream analyses were based on high quality, clean data. Clean data was mapped to the mm9 reference genome using TopHat v2.0.12 (34). The parameters –read-mismatches and –library-type were set to 5 and fr-firststrand, respectively. After that, the expression level FPKM (fragments per kilobase per million mapped fragments) of each gene was measured by Cufflinks (2.2.1) (http://cole-trapnell-lab.github.io/cufflinks/). In this study, the average FPKM was used to represent the expression levels of enhancer target genes.

### Chromatin accessibility of enhancers

The chromatin accessibility of MAE-seq enhancers was determined by DNase-seq and ATAC-seq data. The MAE-seq enhancers that overlapped with peaks from DNase-seq and ATAC-sq were defined as in open chromatin regions, otherwise, they were considered as being in closed chromatin regions. The MAE-seq enhancers in open or closed chromatin regions were classified by BEDTools. Each enhancer was assigned to the one target gene by using the Activity-by-Contact (ABC) model (35). The expression levels (FPKM) of target genes, which were obtained from RNA-seq data, in open and closed chromatin regions were assigned by HOMER and visualized by R (version 4.0.2).

### Escape enhancers detection

To verifying if MAE-seq can detect the ‘escape enhancers’, the Hi-C data (GSE125656) and ChIP-seq data of histone marker H3K9me3 were used. The MAE-seq enhancers overlap with H3K9me3 and have intersection with house-keeping genes were defined as ‘escape enhancer’ according to the report before. The mouse house-keeping were downloaded from https://housekeeping.unicamp.br/ and liftOver was used to transfrom mm10 coordinate to mm9. The overlap between MAE-seq enhancers, genes and loops were detected using BEDTools.

### Defining the category of enhancers

Enhancer candidates were divided into two categories, according to the relationship between MAE-seq loci and known consensus enhancer regions(which included the EnhancerAtlas 2.0 dataset, DNase-seq, ATAC-seq and ChIP-seq of H3K4me1 and H3K27ac). Specifically, the enhancers whose coordinates overlap with EnhancerAtlas 2.0 enhancer regions, DNase-seq, ATAC-seq or ChIP-seq of H3K4me1 and H3K27ac peaks (±2500 bp) were represented as the known enhancers. Otherwise, if the genome positions of enhancers have never been reported in the EnhancerAtlas 2.0 dataset, DNase-seq, ATAC-seq or by ChIP-seq of H3K4me1 and H3K27ac, these loci were assigned to novel enhancers. Additionally, the distance to TSS for these two kinds of enhancers was computed and visualized.

### Functional enrichment analysis of predicted target genes regulated by enhancers

Gene Ontology (GO) analyses of known and novel MAE-seq enhancers were performed by the clusterprofiler package (36) in R with the threshold of FDR < 0.05.

### Analysis of the chromatin interaction related to novel enhancers

The significant chromatin interactions were identified from mESCs Hi-C(data associated with this study are derived from GEO database (GSE125656)) and by HOMER (31) and HiCCUP (32) respectively. The overlap between MAE-seq loci and chromatin interaction anchors was analyzed by BEDtools (27).

### Statistical analysis

We used R (version 4.0.2) (37) and SPSS 19.0 (IBM, Chicago, IL, USA) for all statistical analysis. All results were expressed as the mean ± standard deviation (SD) of the number of experiments.

### Overlapping enhancer and transcription factor motif regions

ChIP peak data for H3K27ac and H3K4me1 were sourced from ENCODE (accessions: ENCFF194TQD and ENCFF158GBZ), with peak coordinates mapped to mm9 using LiftOver. Employing Bedtools intersect, enhancer-ChIP peak overlap was determined, requiring at least 30% fraction overlap. Enhancers overlapping H3K27ac or H3K4me1, alongside randomly selected enhancers, were chosen for downstream analysis. FIMO, utilizing the JASPAR2020_CORE_vertebrates_non-redundant_pfms_meme database, scanned the mm9 genome for potential transcription factor binding motifs. Enhancers were subsequently overlapped with these motif regions, demanding at least 90% fraction overlap. Enumerated and sorted transcription factors associated with enhancer overlap were analyzed. Open chromosomal regions (ATAC peaks, GEO: GSE120376) were integrated using the same overlap methodology.

## Results

### MAE-Seq development

We developed MAE-seq based on random DNA libraries and fluorescence-assisted cell sorting (Figure 1A). To determine the optimal length of the DNA fragments and improve alignment accuracy, we conducted a bioinformatics simulation experiment. Random sequences of different lengths (20–60 bp, 1 bp interval) were extracted from the human genome (hg19). The result showed that, sequences longer than 25 bp had a unique mapping rate of approximately 80%, reaching a plateau stage, except for bowtie2 (Supplementary Figure S1B). Meanwhile, to verify whether the 25 bp DNA length has enhancer function, we intercepted 25 bp from the previously reported enhancer sequence for dual luciferase reporter assay. The results showed that the 25 bp sequence had enhancer activity. This result proves that the 25 bp DNA sequence can play an enhancer role as an independent functional unit. (Figure 1B). Thus, our results demonstrate that 25-bp fragments have advantages in both alignment accuracy and sensitivity, making them suitable for MAE-seq system development. 25-bp fragments are used to construct random DNA libraries and subsequently screen active enhancers.

In brief, 25-bp DNA random fragments are constructed into a ‘classic’ MPRA plasmid vector (38) with a fluorescent reporter (Supplementary Figure S1A) and then transfected into cultured cells (70%-80% confluent). If the insert fragment has enhancer activity, then it will interact with the mini promoter to make the downstream mCherry express red fluorescence. In the MAE-seq, the plasmid transfected cells are divided into two groups. One is used for flow fluorescence sorting. The enriched fluorescence cells are used for DNA extraction and later specific amplification. These fragments are applied for high-throughput sequencing (termed ‘output library’, Supplementary Figure S1C). These sequenced 25-bp fragments are considered to contain a high-ratio positive signal. To mitigate potential background noise and control for factors like multiple plasmids entering a single cell, the input library is constructed directly from the other group of transfected cells, without flow fluorescence sorting and sequenced. This is considered a control library. Similar to the ChIP-seq analysis (12), we design a model (see methods) based on Poisson distribution with input and output datasets to capture the true enhancer signal. For example, in mESCs, 5.5 million positive candidates were obtained from the output library. After model filtering, 626879 valid enhancer candidates are identified (Supplementary Table S5). To evaluate the model's efficiency, we use enhancer histone markers (H3K4me1) to determine the result. It shows that although the number of positive candidates drops sharply (from 5521717 to 626879, down ∼89%) after filtering, however, the overlap with H3K4me1 only decreased by 13% (from 123 376 to 107 271). This indicates the filtering model works efficiently, and the identified enhancer candidates will be used for the further analyses.

Using MAE-seq, we identified a total of 626 879 and 554 826 (Supplementary Table S6) 25-bp enhancers in mESCs and HEK 293T. To validate their enhancer activity, 40 MAE-seq sites from mESCs and HEK 293T were selected for dual luciferase assay(Supplementary Table S1). Remarkably, at least 80% of the MAE-seq sites showed enhancer activity. (Figure 1D, Supplementary Figure S1E). By constructing these loci into reporter vectors containing mCherry fluorescent genes and transfecting cells, fluorescence expression could be observed under the microscope. (Figure 1C, Supplementary Figure S1D,G). Further, we selected 20 sequences in mESCs that were from non-fluorescent cell populations, and only 1 sequence was shown to have enhancer activity by the dual-luciferase reporter system (Supplementary Figure S1F), demonstrating the effectiveness and reliability of the MAE-seq data.

### Epigenetic characteristics and chromatin accessibility

**Figure 2.**
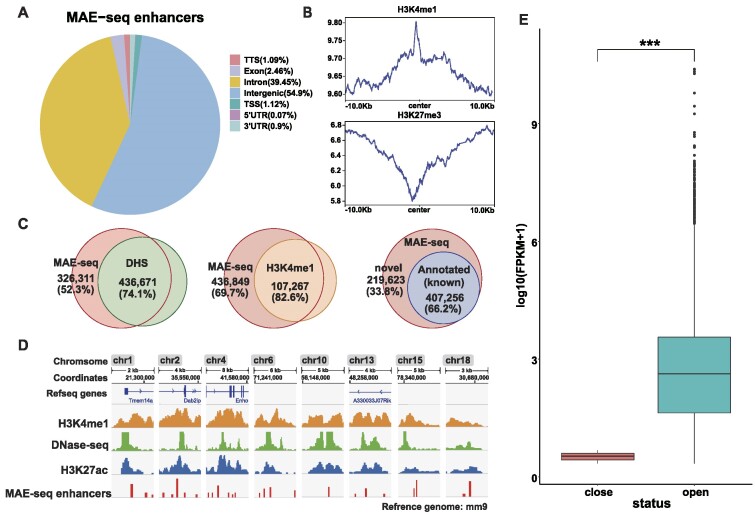
Genomic annotation of MAE-seq enhancers. (**A**) Genomic distribution of MAE-seq enhancers. In mESCs, 626879 million loci have been identified by MAE-seq. Most of them are in non-coding regions such as intergenic and intron. (**B**) The enrichment of H3K4me1 and H3K27me3 marks around the MAE-seq loci. (**C**) The percentage of enhancer epigenetic markers(H3K4me1), DNase-seq and EnhancerAtlas 2.0 data base covered by MAE-seq sites. (**D**) Distribution of 25bp MAE-seq enhancers in histone modification regions(H3K27ac, H3K4me1, DNase-seq) of 8 chromosomes. (**E**) Expression of nearest genes related to MAE-seq enhancers which are in open and closed chromatin regions. Open, represent DHS; closed, represent none of DHS. *** *P*< 0.001.

To comprehensively annotate and analyze our MAE-Seq datasets, we realize there were more diverse and experimental datasets generated in mESCs compared to HEK 293T cells, making them more suitable for annotation and interpretation purposes (Epigenetic characteristics analysis of HEK 293T cells is shown in Supplementary Figure S6). Therefore, our following analysis focuses on interpreting mESC datasets. In mESCs, we identified 626 879 enhancers through MAE-seq. We analyzed the genomic distribution of MAE-seq enhancers. The results show that over 90% of enhancers are located in intergenic or intron regions, which is consistent with predictions based on chromatin features (39,40) (Figure 2A).

To further validate their genomic features, we performed a joint analysis with both known enhancer and repressed epigenetic data. The result showed that H3K4me1 (41) was significantly enriched at these loci, while the signals of the repressed chromatin mark, H3K27me3, vanished at these loci in mESCs (Figure 2B). To annotate these loci, we performed an overlap analysis with different active genomic datasets. First, with the H3K4me1 enhancer marks, we found that 82.6% of them had MAE-Seq signals (Figure 2C). It is consistent with the enhancer feature of the MAE-Seq loci. Next, we overlapped them with the active chromatin datasets, the DNase I hypersensitive sites (DHSs), and we found that 74.1% of the DNase-seq sites can be annotated by MAE-seq loci (Figure 2C). This indicated MAE-Seq loci mainly located at the open chromatin region (42). Lastly, we compared them with the Mouse Enhancer Atlas 2.0 database (43), which overlaps with MAE-Seq loci. The result indicated that about 66.2% of the MAE-seq loci could be annotated (Figure 2C). Together, these confirm that the MAE-Seq loci are enriched with enhancer and active chromatin signals, and demonstrate the informativeness and reliability of our data. In addition, the ChIP-Seq binding sites of various ESC-specific transcription factors (TFs), such as Sox2, Nanog and Oct4, had also been used to do the annotation, and the results are consistent with active genomic datasets (Supplementary Figure S2A). Again, these genomic datasets suggest our MAE-seq sites are informative and enriched with enhancer or active chromatin signals (Figure 2D). These further confirm the precision of the strategy.

Previous research on transcription networks and REs primarily focused on open chromatin regions of the genome. To address this, we analyzed the distribution of MAE-seq enhancers in both open and closed chromatin regions in mESCs. As expected, we found 74.27% of known MAE-seq enhancers presenting in open chromatin regions, which is 2.9-fold the percentage of enhancers in the closed chromatin regions (Supplementary Figure S2B, Supplementary Table S2). Next, following Cosmas D. Arnold's study (44), we divide MAE-seq enhancers into open enhancers and closed enhancers according to whether they appear in open chromatin regions or not and assess the expression levels of their nearest genes. The results reveal that the target gene expression levels of closed enhancers are significantly lower (5.23-fold) than those of open ones (Figure 2E). These findings imply that these closed enhancers have enhancer sequence potential, but may be silenced by the local endogenous chromatin environment.

In conjunction with the Hi-C data, we found some enhancers in heterochromatin (H3K9me2 and H3K9me3 markers) that interact with mouse housekeeping genes, including Rrn3, Atf7ip, Mta3, and so on. At the sites where we interact with these genes, luciferase experiments have shown that they have high enhancer activity. (the average activity intensity is more than 400% higher in the control group) (Supplementary Figure S2C, Supplementary Table S2). The properties of these enhancers are similar to those of the ‘escape enhancers’ previously reported in the literature (45). This suggests that not all the enhancers would be silenced in their endogenous contexts. Still, quite a few enhancers retained their activity in closed chromatin regions. Their activities may be independent of the environment of endogenous chromatin. Additionally, we explored the relationship between enhancer activity intensity and histone markers (DNase, H3K27ac and H3K4me1). Through luciferase experiments, we found that the luciferase intensity with enhancer histone mark sites was not significantly higher than that without histone mark sites, indicating that these chromatin signatures are not a good predictor of enhancer activity strength (Supplementary Figure S2D, Supplementary Table S2).

### Determining the regulatory function of 25-bp fragments

**Figure 3.**
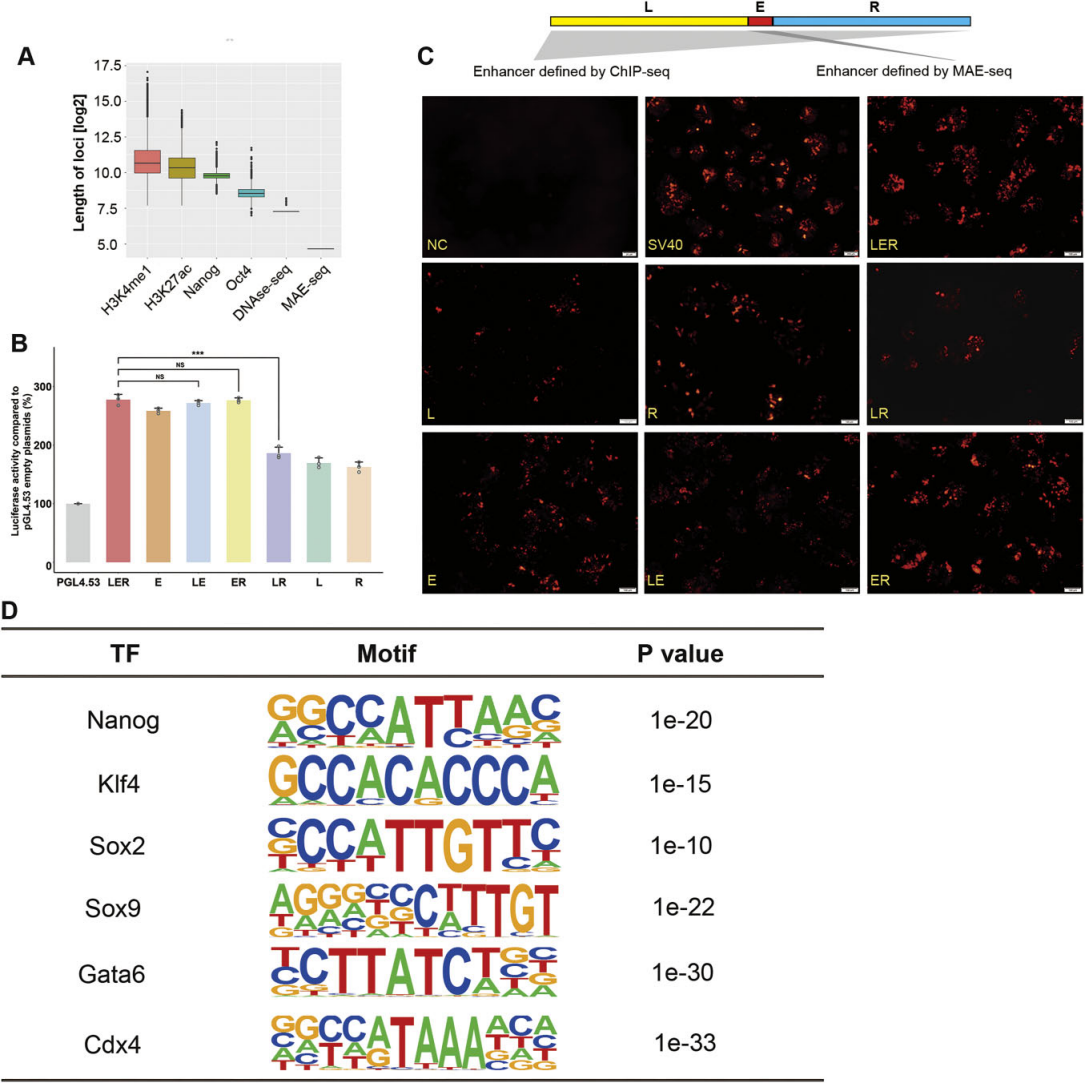
Functional validation of MAE-seq enhancers. (**A**) The average length of enhancers annotated by epigenetic modification or transcription factor binding compared with the length of the MAE-seq enhancer. Data of sequence length is converted to log_2_. (**B**) Differential analysis of enhancer activity of different fragments in ‘LER’ experiment by luciferase. L, left flanking sequence; R, right flanking sequence; E, 25bp enhancer. empty pGL4.53 plasmid was used as the control for baseline luciferase activity. The y axis represents the percentage of luciferase activity compared to pGL4.53 empty plasmids in the respective cells (*n* = 3 biological independent samples; bars show mean value ± s.e.m.; ns: non-significant, *** *P*< 0.001, calculated using *t*-test). (**C**) mCherry fluorescence expression profile of different fragments in ‘LER’ experiments after transfection of mESCs. Different fragments were constructed into the reporter vector with mCher gene and transfected into cells, and the fluorescence expression was observed after 48 h. NC, empty reporter vector; SV40, SV40 enhancer; L, left flanking sequence; R, right flanking sequence; E, 25 bp enhancer. (**D**) The mESCs specific transcription factor motifs identified from mESCs MAE-seq data.

The span of REs identified by MAE-seq is much shorter compared to those defined by ChIP-seq (H3K4me1, H3K27ac, Oct4 and Nanog) and DHS-seq (25-bp versus ∼600bp) (Figure 3A). To determine whether such a short sequence can function efficiently as the previously defined REs, we randomly selected five ChIP-seq or DNase-seq binding loci that overlap with the MAE-seq sequences (Supplementary Table S3). Firstly, we divided each binding locus into three fragments, referred to as ‘L’, ‘E’ and ‘R’. The fragment, which overlapped with a fragment from MAE-seq data is referred to as ‘E’, the fragment to its left is referred to as ‘L’, and the fragment to its right is ‘R’. We then synthesized these sequences using different combinations. As a result, for locus 1, when ‘L’ is missing, the remaining fragments (E&R) have no significant change in activity change. When the ‘R’ is missing, the remaining fragments (L&E) have no significant change in activity. As expected, when the ‘E’ is missing, the activity of all remaining combinations (L, R and L + R) shows a sharp decrease or disappears in the experiments (Figure 3B, C). We have performed 3 biological replicates for each of these experiments. Out of those 5 loci, 4 have shown similar results. This implies that these regulatory activities are heavily dependent on these 25 bp MAE-Seq defined fragments. Similar results were also found in HEK293T (Supplementary Figure S3A,B). Together, these results suggest that ChIP-seq/DHS-Seq defined loci have veiled sequence regions and need further annotation.

Enhancers typically require binding TFs to perform their function (46). To further verify whether the MAE-seq data can enrich transcription factors with these 25-bp fragments, we used the findMotifsGenome.pl program in the software HOMER(31) to perform motif analysis with these 25-bp sequences, and the set parameter is -size (the default parameter is not set). As expected, these fragments could significantly enrich embryonic stem cell specific transcription factor binding sites, such as Sox2, Klf4 and Nanog (*P* value < 0.001) (Figure 3D). In summary, these imply that these 25 bp fragments could be used for motif analysis. Together, the defined 25-bp fragments could perform the similar regulatory functions as the ∼270 bp chromatin-based loci and could also be used for bioinformatics analysis.

### Identification and verification of novel candidates

**Figure 4.**
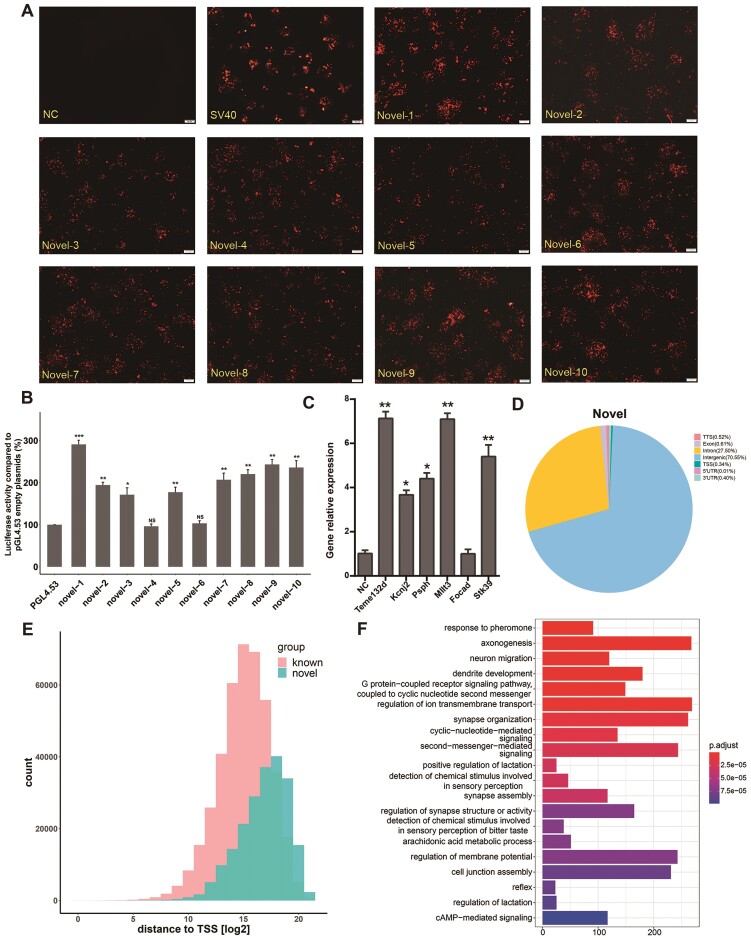
Genomic annotation and Functional validation of novel enhancers discovered by MAE-seq. (**A**) mCherry fluorescence expression of novel enhancers. Mouse enhancers annotated in EnhancerAtlas 2.0 database are used for classification of MAE-seq enhancers. 35.3% of MAE-seq enhancers that are not covered by the database are classified as novel enhancers. Ten of them were randomly collected and constructed into a reporter vector with mChery gene, and the fluorescent expression was observed 48 h after transfection of cells. NC, empty reporter vector; SV40, SV40 enhancer; Novel-1 to Novel-10, ten novel enhancers. **(B)** The enhancer activity of the 10 novel enhancers in (A) was quantified using a dual luciferase reporter system. empty pGL4.53 plasmid was used as the control for baseline luciferase activity. The y axis represents the percentage of luciferase activity compared to pGL4.53 empty plasmids in the respective cells (*n* = 3 biological independent samples; bars show mean value ± s.e.m.; ns: non-significant,**P< 0.01, ***P< 0.001,calculated using *t*-test). (**C**) The regulation effect of novel enhancers on the transcription level of the target genes. Six novel sites are selected for endogenous activation and the changes in the transcription level of their target genes are detected by QPCR (* *P*< 0.05; ** *P*< 0.01, calculated using *t*-test). (**D**) Genomic distribution of novel enhancers. (**E**) Statistics of distance to TSS of novel enhancers. Red column represents known mouse enhancers; the blue column represents novel enhancers. (**F**) GO enrichment analysis of nearest genes related to novel enhancers.

EnhancerAtlas 2.0 is a comprehensive database that contains an atlas dataset from 241 cells, and tissue types in mice (47). Notably, 35.03% of MAE-seq enhancer loci have never been detected by DNase-seq or ChIP-seq (H3K27ac, H3K4me1, ESC-specific TFs) in any mouse cell type or tissue. These 35.03% MAE-seq sites as novel enhancers. To verify whether these novel sequences have enhancer activity, we randomly selected 10 novel enhancers and constructed them in a Luciferase reporter vector (Supplementary Table S1). The result showed that 80% of sites have enhancer activity (Figure 4B). They were constructed into a vector containing the mini promoter of the mCherry gene, and we could observe the expression of the fluorescent gene 48 h after transfection cells (Figure 4A). To further verify the effect of these elements on gene expression, we randomly selected six novel enhancers, using the dcas9-SunTag-VP64 system (48) to precisely activate these sites in the endogenous environment, and then the changes of distal regulatory genes expression were detected by QPCR combined with Hi-C data (Supplementary Table S4). The expression levels of the five distal regulatory genes are significant upregulated (Figure 4C). We also examined potential off-target sites at each location, and the sequencing result showed that there are no mutations at these sites. These results are consistent with the previous finding that genes can be regulated by distant enhancers (49). Also, it implies that these novel elements do play critical roles in gene transcription regulation in living cells via distal associations. Therefore, this implies that MAE-seq is able to capture the omitted regulatory genomic information that is not elucidated by previous methods.

To further characterize these novel elements, we analyzed their genomic distribution pattern. Similar to the known markers, the majority (70.55%) of these novel candidates are distributed to distal intergenic regions, followed by introns (Figure 4D). Next, we analyzed the distance to the nearest gene TSS of novel enhancers. Different from known ones, more novel enhancers prefer to be found in the intergenic region, far away from the TSS (Figure 4E). This suggests that these novel enhancers enact remote regulation of the target gene's expression. We tested this speculation by analyzing novel enhancers with Hi-C and Micro-C datasets (50). The results show that 122 199 novel enhancers may be involved in the formation of the chromatin loop, accounting for 55.40% of the total number of novel enhancers (Supplementary Figure S4A). Additionally, the average span of these interactions reaches 126 kb, implying that the distal interaction with promoters mediates the function of novel enhancers. To explore the roles of these types of enhancers in the biological process, GO analysis is conducted. The result shows that most of the nearest genes related to known enhancers play roles in the transport of ions and proteins across membranes, such as calcium ion homeostasis, localization, and transport of proteins (Supplementary Figure S4B). In contrast, genes linked to novel enhancers were significantly enriched in biological processes related to the neural correlates, such as axonogenesis and synapse organization (Figure 4F).

In a recent study, it was demonstrated that the majority of noncoding regions in the human genome lack annotated elements and lack epigenomic or protein binding signals. Interestingly, they selectively knocked out a region called hub_22_7, which significantly affected the cell phenotype (7). Among the sites they verified, 444 (46.25% of 960) coincided with the MAE-seq sites of human cells. In order to verify the function of these sites, we selected nine sites for luciferase verification in HEK 293T cells. The results showed that these sites showed enhancer activity (Supplementary Figure S4C). Further experiments showed that activation of these sites could significantly increase the expression level of their target genes (Supplementary Figure S4D). This discovery further emphasizes the ability of MAE-Seq to define novel enhancers from a different perspective. It suggests that the new tools, such as our MAE-Seq, are increasingly required for precise and functional annotation of regulatory elements throughout the genome.

### Multiple refined elements hierarchically regulate *Cdh1* expression

**Figure 5.**
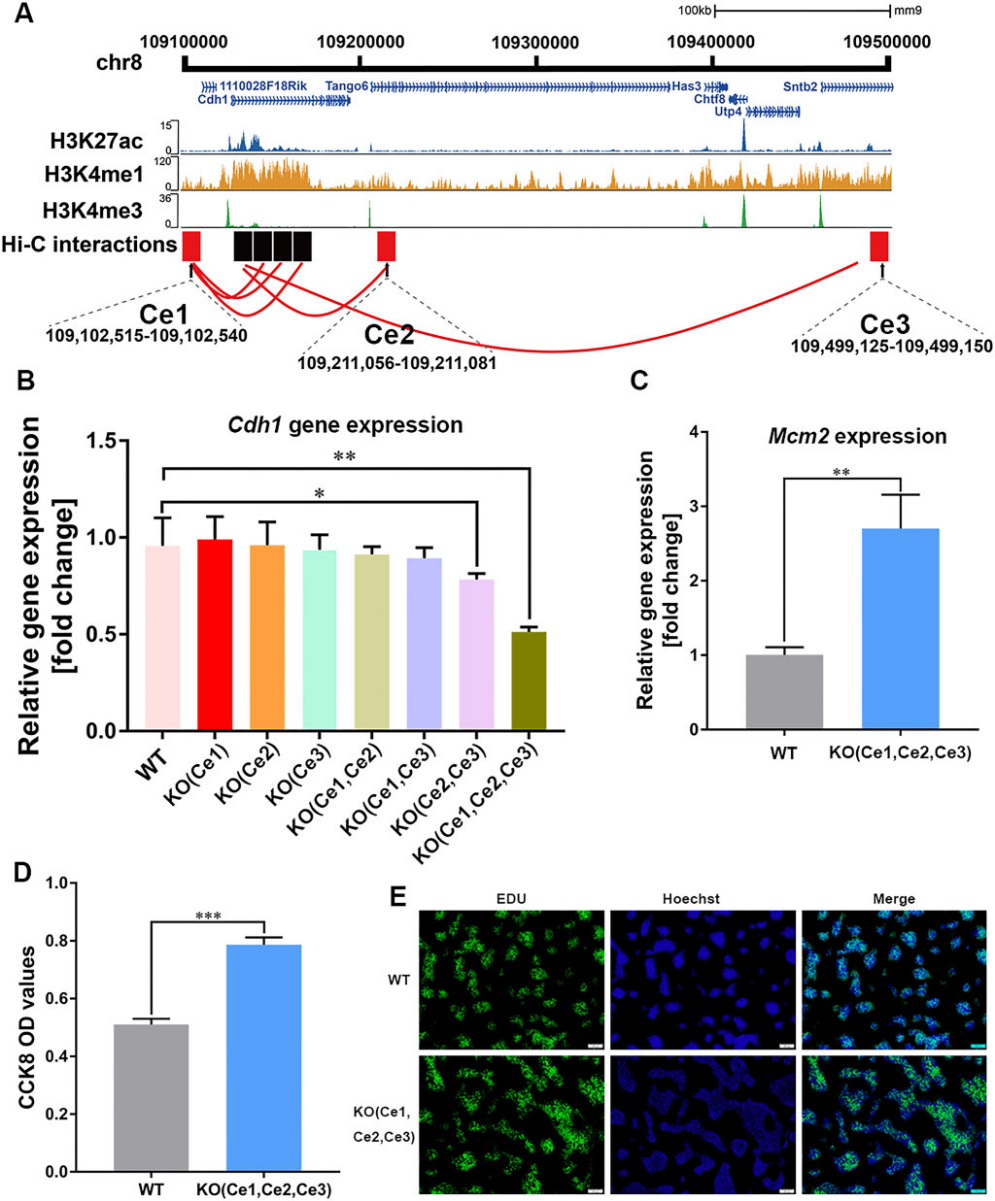
Endogenous functional validation of MAE-seq enhancers targeted to *Cdh1* gene. (**A**) Chromatin interaction between the *Cdh1* gene and the MAE-seq enhancers (Ce1, Ce2 and Ce3). Red represents the location of the enhancers, and black represents the region of the *Cdh1* gene that interacts with the enhancer. (**B**) Expression of *Cdh1* gene in enhancer knockout cells. Expression level of *Cdh1* gene is significantly downregulated in Ce2 & Ce3 double knockout and Ce1& Ce2 & Ce3 triple knockout cell compared with wild type cell. Each group has three technical replications. * *P*< 0.05; ** *P*< 0.01. (**C**) Mcm2 expression of triple knockout cell. ns: not significant; ** *P*< 0.01,WT, wildtype cell; KO, triple knockout cell. (**D**) CCK8 detection of triple knockout cells. *** *P*< 0.001, WT, wildtype cell; KO, triple knockout cell. (**E**) EDU proliferation experiment of triple knockout cell. WT, wildtype cell; KO, triple knockout cell.

To evaluate the potential impact of MAE-Seq enhancers on cell phenotype, we focused on the gene of interest, *Cdh1*, for further validation. Direct interactions between three MAE-seq enhancers (*Cdh1* enhancer1(Ce1), *Cdh1* enhancer2(Ce2) and *Cdh1* enhancer3(Ce3)) (Supplementary Table S4) and the promoter of the *Cdh1* gene were found in Hi-C datasets (Figure 5A). Among them, Ce1 and Ce3 are known enhancers, and Ce2 is a novel enhancer. All these interaction loci have histone mark activities (Figure 5A). To explore the biological function, CRISPR/Cas9 system (Supplementary Figure S5A) was employed to assess their requirement for gene expression in genomic loss-of-function models. We successfully generated homozygous (-/-) knockout mESC lines for single knockout, double knockout, and triple knockout. To investigate whether these three enhancers can affect cell phenotype individually, they are deleted individually in mESCs (Supplementary Figure S5B). We found that when knocking out any single one of them, the transcription level of the *Cdh*1 gene did not change significantly (Figure 5B). To amplify the effect, double knockouts of different enhancer combinations (Ce1&Ce2, Ce2&Ce3 and Ce1&Ce3) are conducted. Our result shows that only Ce2&Ce3 double knockout cells slightly downregulate the expression of the *Cdh1* gene (Figure 5B). Notably, when we knocked out all three enhancers, the transcription level of Cdh1 decreased significantly (Figure 5B). These results imply that proper expression needs coordination, and it may be hierarchically regulated.

In a previous study, downregulation of the *Cdh1* gene will affect the proliferation of cancer cells (51). We explored this phenomenon in mESCs with the Ce1, Ce2 and Ce3 triple knockouts. When we inoculated the same density of wild-type and knockout cells in a 6-well plate for 48 h, we observed a significantly higher number of cells in the knockout cell line compared to the wild-type (Supplementary Figure S5C). To detect the cell cycle difference more directly between the knockout cell line and the wild-type cell line, we tested the expression levels of cell cycle-related genes *Mcm2* and *Pcna*. The expression levels of the knockout cell lines’ *Mcm2* and *Pcna* were significantly upregulated (Figure 5C, Supplementary Figure S5D). Next, we used the CCK8 method to detect the proliferation ability of the knockout cell line more directly, and the result showed that the number of viable cells in the knockout cell line was significantly higher than that of the wild type (Figure 5D). To observe the difference in the number of DNA replication cells more intuitively, we used EDU to stain the two cell lines. It shows that the number of cells stained by EDU in the knockout cell line was significantly higher than that of the wild-type (Figure 5E). These results reveal that the novel enhancers identified by MAE-seq play an important role in regulating gene expression and cell phenotype.

Overall, with the high-resolution interactome data and MAE-Seq loci, we were able to identify the *Cdh1*′s multiple enhancers-one promoter regulation pattern. This knowledge may represent a critical step towards understanding its precision biology.

### Identification of cell-specific enhancers and accurate annotation of super enhancers

**Figure 6.**
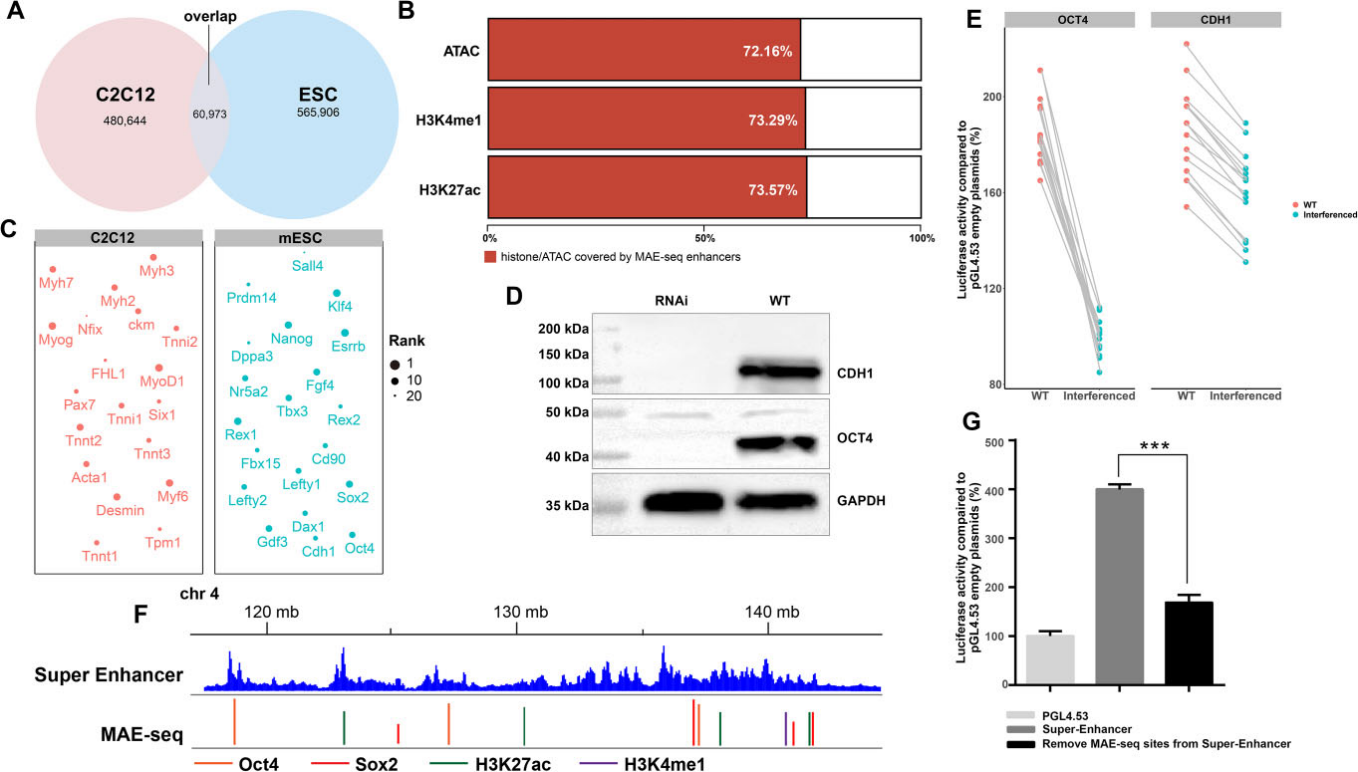
MAE-seq can identify cell-specific enhancers and accurately annotate super enhancers. (**A**) The overlap between C2C12 and MAE-seq enhancers identified by mESCs. (**B**) The coverage percentage of MAE-seq enhancers identified in C2C12 to ATAC-seq, ChIP-seq (H3K3me1, H3K27ac) data in C2C12. (**C**) The types of single specific TFs bound to enhancer sequences in C2C12 and mESCs. The size of the point represents the ranking of the degree of enrichment. (**D**) Cas13d interfered with the expression levels of corresponding proteins in cells after 48 h of OCT4 and CDH1 genes. WT, wildtype cell; RNAi, Cas13d RNAi cell. (**E**) The changes of enhancer activity in corresponding cells after OCT4 and CDH1 interference. WT, wildtype cell; interferenced, Cas13d RNAi cell. (**F**) Comparison of peaks of Super-Enhancer and MAE-seq sites. A Super-Enhancer of chr 4 is shown in the figure. (**G**) The activity comparison between super enhancer and super enhancer knocking out MAE-seq site; empty pGL4.53 plasmid was used as the control for baseline luciferase activity. The y axis represents the percentage of luciferase activity compared to pGL4.53 empty plasmids in the respective cells (*n* = 3 biological independent samples; bars show mean value ± s.e.m.; ns: non-significant,***P< 0.001,calculated using *t*-test).

To assess the capability of MAE-seq in recognizing cell-specific enhancers, we applied this technique to identify 554826 enhancers within C2C12 cells(Supplementary Table S7). Comparative analysis involving these enhancers and those found in mESCs revealed that merely 12.69% of C2C12 enhancers overlapped with mESCs enhancers (Figure 6A). Impressively, the remaining non-overlapping enhancers encompassed >70% of cell-specific ATAC-seq, H3K4me1, and H3K27ac ChIP-seq data (Figure 6B). The heightened resolution of 25 bp enhancers facilitated precise one-vs-one analysis of enhancers and transcription factors (TFs), which contrasted with longer enhancer sequences. Notably, a substantial portion (61.1%) of MAE-seq enhancers in mESCs bound solely to a single TF on their sequence (Supplementary Figure S7A), in contrast to longer sequences like super enhancers (Figure S7B). Strikingly, analysis of TF types bound by these enhancers unveiled a prevalence of cell-specific TFs, including Klf4, Esrrb, Sox2 and Oct4 (Figure 6C). Parallel observations were evident in C2C12 cells, featuring specific TFs such as MyoD1 and Myog (Figure 6C). Expression levels of these enhancers target gene notably exceeded the average level (Figure S7C), accentuating their functional relevance. To corroborate the impact of cell-specific TFs on enhancer activity, we employed Cas13d (52) to target Oct4 and Cdh1. Notably, fluorescence quantification demonstrated their substantial downregulation (Figure S7D), an observation confirmed by diminished protein expression (Figure 6D). Dual luciferase assays corroborated the attenuation or disappearance of enhancer activity upon interference (Figure 6E). Intriguingly, the most enriched TFs within mESCs-specific enhancers were Esrrb and Klf4, aligning with superenhancer findings (21). Notably, the integration of superenhancer sites and MAE-seq sites affirmed the precise annotation of TF and histone binding sites within superenhancers (Figure 6F). Furthermore, removal of these MAE-seq sites from superenhancers led to a rapid decline in their activity (Figure 6G), highlighting MAE-seq's accuracy in annotating superenhancers, while underscoring that superenhancers comprise a compilation of multiple cell-specific MAE-seq enhancers.

### Differential cofactor dependencies linked to enhancer chromatin environment

**Figure 7.**
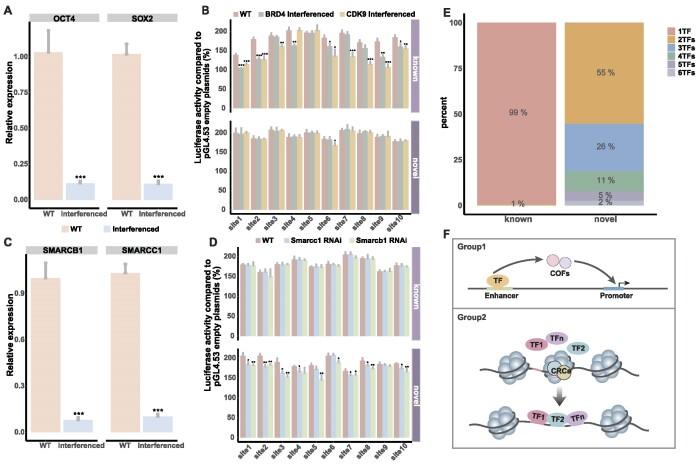
Differential cofactors are related to the chromatin environment of the enhancer. (**A**) After 48 h of small molecule inhibitors interfering with *BRD4* and *CDK9* genes, QPCR was used to detect the changes of corresponding gene expression. ***P< 0.001, calculated using *t*-test. (**B**) The changes of known and novel enhancer activity after *BRD4* and *CDK9* interference. The activity of 10 novel and known enhancers was detected in the cells after BRD4 and CDK9 interference. WT, wildtype cell. (**C**) After 48 h of small molecule inhibitors interfering with *SMARCC1* and *SMARCB1* genes, QPCR was used to detect the changes of corresponding gene expression. ***P< 0.001, calculated using *t*-test. (**D**) The changes of known and novel enhancer activity after *SMARCC1* and *SMARCB1* interference. The activity of 10 novel and known enhancers was detected in the cells after BRD4 and CDK9 interference. WT, wildtype cell. (**E**) The number of TFs combined on the known and novel enhancer sequences is shown in the graph as a result of ≤6 TFs. (**F**) Cofactors and TFs regulate known and novel enhancer regulatory mechanism models.

The orchestration of enhancer-mediated transcription necessitates cofactors. To discern differences in novel and known enhancer reliance on cofactors, we employed small molecule inhibitors to target CDK9 and BRD4 genes. Quantitative PCR results evidenced substantial downregulation 48 h post-interference (Figure 7A). To probe the impact on enhancer activity, dual luciferase assays demonstrated significant reductions in 90% of known enhancers (Figure 7B), with diverse CDK9 and BRD4 perturbation effects. In contrast, only the sixth novel enhancer experienced CDK9-mediated activity reduction, suggesting context-dependent responses (Figure 7B). Given the enclosed chromatin state of novel enhancers, we probed the influence of chromatin environment on cofactor dependence. The results of interference with SMARCC1 and SMARCB1 showed that the expression levels were down-regulated (Figure 7C). In terms of the effect on enhancer activity, the activity of novel enhancers decreased significantly or disappeared, while the activity of known enhancers was not affected (Figure 7D). These findings suggest that novel enhancers depend on the CRCs family, while known enhancers rely on the COFs family, contingent on chromatin state. Additionally, novel enhancer sequences exhibited significantly higher TF binding compared to known enhancers (Figure 7E). These observations culminate in a proposed model whereby known enhancers bind a TF and subsequently recruit COFs for target gene interaction. Notably, within closed chromatin regions, collaboration between TFs drives the CRCs family to compete with nucleosomes for DNA binding, ultimately binding to novel enhancer sequences (Figure 7F), akin to the ‘billboard’ model of enhancer collaborative binding (53).

## Discussion

In the past few decades, scientists have made significant efforts in defining regulatory elements (REs) across the genome, employing experimental and bioinformatics analyses. However, most experimental methods are based on chromatin, and struggle to precisely pinpoint the loci of these elements. The length of defined REs is typically several hundred base pairs (∼270 bp in average) (54). Recently, the length of STARR-Seq defined REs has been analyzed and is similar to the results of the above methods. In contrast, the motif analysis of these binding sites has revealed that the core sequences of these REs are typically 6–14 bp long. However, studies have also indicated that these motif sequences might not function independently, as their activities depend heavily on the flanking sequences at both ends (55,56). These flanking sequences can facilitate efficient transcription factor binding or modulate binding affinity to influence gene transcription regulation.

A recent study investigated the activity of REs with different fragment lengths and found that long fragments, besides being less repetitive, displayed advantages in enhancer activity. The researchers speculated that long fragments may provide more position for transcription factor binding (19). In contrast, our data present a different perspective. We propose that the flanking sequences of some long fragments may also bind some transcriptional repressors and influence enhancer activity. We believe that within long fragments, there exists a core fragment that binds the most crucial transcription factors. This core fragment acts as a functionally independent element and this core fragment is a functionally independent element, and its deletion directly affects the activity of the entire sequence. A study by Jason *et al.* also mentioned that deletion of the middle 354bp from a long fragment (678-bp) resulted in the loss or reduction of activity for the entire fragment. In our study, we observed a similar effect with a 25-bp deletion through the ‘LER’ experiment and demonstrated by knockout experiment that this 25-bp deletion can affect cell phenotype. These experimental results support our hypothesis to some extent, bringing us closer to precise genome annotation.

Over the past two decades, ENCODE Project and Epigenome Roadmap Consortiums have invested tremendous efforts in generating comprehensive predictive annotations of REs located in non-coding regions of the genome (57,58). According to the latest results, 2.1 million REs (enhancers) have been proposed or predicted across the human genome, ∼567 million bp (18% of the whole genome, 40% of non-repetitive genome sequences) in total (59). However, with our MAE-seq data, while these annotations cover most of the known REs, they suggest that only approximately 52.5 million base pairs are functional sequences. The remaining ∼514.5 million base pairs may have other potential functions that need further investigation.

At present, most putative REs are identified via chromatin-based assays, followed by high-throughput sequencing such as ChIP-seq, DNase-seq and FAIRE-seq (12–14). Usually, these candidates need validation experiments to determine their specific function through various analyses. Recent studies have indicated that such RE annotation might be incomplete. For example, sequences labeled with P300 and H3K27ac show a high false positive rate on enhancer validation, and fragments without binding capacity can also show regulatory activities (60). This has also been observed that histone marks previously associated with active genes do not directly cause transcriptional activation(61). In our study, we surprisingly find that a considerable number of our identified enhancers (219623, ∼35.03%) have no signal on either histone or annotation, based on current knowledge. This finding aligns with a recent study (7) and our validations demonstrated that most of these novel loci exhibited regulatory activity. It suggests that epigenetic modifications alone are insufficient for genome-wide RE annotation. Instead, it likely depends heavily on the local chromatin environment, three-dimensional (3D) genome folding, and the relative concentrations of various downstream effector proteins (62–64). Our results indicate that numerous sequences might function in this manner.

The biological function of non-coding sequences has always been an intriguing subject. Unfortunately, there are few reports in this area. For example, the deletion of the enhancer sequence of Sox9 can lead to gender reversal in mice (65). A recent study reported that non-coding sequences without any epigenetic characteristics can affect cell apoptosis (7). In our study, we performed knockout experiments on three short enhancers interacting with *Cdh1*, two of which were known and one was *de novo*(lacking epigenetic characteristics)identified in our study. CRISPR/Cas9-mediated knockout experiments confirmed that these sequences can regulate cell phenotype. This implies an elegant ‘multiple-enhancer-one promoter’ regulation model. Moreover, we notice that there are more than 2.1 million known enhancers and only 22000 genes annotated in the human genome (59,66). Based on our findings,, the number of REs could be expected to increase significantly. Therefore, our data support the prevalent of ‘multiple elements-one target gene’ pattern (67,68) that surpasses initial expectations. In this study, we used *Cdh1* as a model to further explore this hypothesis. We found that among the 3 tested enhancers, none of them was redundant in single enhancer knock-out experiment (69). This study verifies the involvement of the novel *Cdh1* enhancer in hierarchical transcriptional regulation and confirms the biological function of short non-coding sequences. It might represent a prevalent model for precise transcription regulation.

Cell-specific transcription factors (TFs) are hallmarks of cellular identity, and prior enhancer studies often faced challenges in achieving precise ‘one-vs-one’ enhancer-TF analysis and identifying enhancers exclusively binding to cell-specific TFs. Remarkably, most MAE-seq enhancer sequences exclusively bind a single TF, enabling identification of numerous cell-specific TF-binding enhancers. Astonishingly, these enhancers effectively annotate functional regions within super enhancer sites. Although our MAE-seq approach doesn’t encompass the entire genome, and thus did not annotate all enhancers (231) reported in the literature (21), this direction might remain a potential avenue for the future work. The role of enhancers necessitates the recruitment of cofactors by TFs. While previous studies highlighted the dependence of enhancers on COFs family cofactors, our study indicates that epigenetic variations underlie differential cofactor (70) requirements for novel and known enhancers. This implies intricate regulatory mechanisms governing enhancers within the genome.

MAE-seq stands out as an innovative and robust method for the precise annotation of functional enhancers within the genome. Its versatile nature enables a wide spectrum of applications in various biological domains, encompassing fields like comparative genomics, disease biology, and synthetic biology. Nonetheless, it is imperative to recognize the limitations inherent in MAE-seq. This method operates within a plasmid system, preventing the study of enhancers in their native chromatin context. Researchers should be mindful of this constraint when planning experiments and interpreting the results. Furthermore, it's worth noting that MAE-seq can be relatively resource-intensive in terms of both cost and time. Nevertheless, the advantages of MAE-seq, such as its high resolution and accuracy, often outweigh these limitations.

While ChIP-seq and other tools can identify genome-wide REs, their spans typically range from several hundred base pairs or longer. Therefore, in the capture of most known REs, MAE-seq is a useful method for precisely identifying active REs, including those without epigenetic marker. It enables re-evaluating the genome annotation and refines our understanding of REs, great facilitating the experimental design for gene function analyses. Hence, MAE-seq provides a valuable tool for unraveling or precisely annotating the ‘dark matter’ across the genome.

## Supplementary Material

gkad1129_supplemental_files

## Data Availability

All the sequencing data and processed data of this MAE-seq research are available on Gene Expression Omnibus(GEO) with GEO accession: GSE193494; https://www.ncbi.nlm.nih.gov/geo/query/acc.cgi?acc=GSE193494.
