# Supplementary material for: MAE-seq refines regulatory elements across the genome
Source: Nucleic Acids Res. 2023 Dec 1;52(2):e9. doi: 10.1093/nar/gkad1129 (PMC10810209; doi:10.1093/nar/gkad1129)
Supplement: gkad1129_supplemental_files [file gkad1129_supplemental_files.zip › Supplementary Material-N24.pdf]

Supplemental files for

# MAE-seq refines regulatory elements across the genome

This files includes:

MAE-seq screening vector.

Figure S1 Supporting materials related to MAE-seq development.

Figure S2 Epigenetic characteristics and chromatin accessibility of MAE-seq loci.

Figure S3 Activity validation of MAE-seq enhancers with or without left or right flanking sequences in HEK 293T cell.

Figure S4 Participate in the ratio of chromatin loop novel enhancers and the GO analysis of known enhancers.

Figure S5 Ce1 to Ce3 knockout results supplement to figure 5.

Figure S6 Epigenetic characterization and taxonomic analysis of HEK 293T.

Figure S7 The number of TFs bound to long sequences and the expression of target cofactors after interference with cells.

Table S1 Location of randomly verified MAE-seq enhancers in different cells.

Table S2 Public data sources used in this study.

Table S3 The genomic locations of MAE-seq enhancers and long-segment enhancers in the "LER" experiment.

Table S4 The enhancer target gene location for verification, sgRNA for CRISPR activation/knockout experiments, and QPCR detection primers.

Table S5 The MAE-seq enhancer location information ( mm9 reference genome, known and novel classifications have been performed on it ) identified in mESCs.

Table S6 The MAE-seq enhancer location information ( hg19 reference genome, known and novel classifications have been performed on it ) identified in HEK 293T.

Table S7 The MAE-seq enhancer location information ( mm9 reference genome, known and novel classifications have been performed on it ) identified in C2C12.

MAE-seq screening vector sequence:

CTGCATAATGAAAGACCCCACCTGTAGGTTTGGCAAGCTAGCTTAAGTAACGCCATTTTGCAAGGCATGGAAAAATA
CATAACTGAGAATAGAAAAGTTCAGATCAAGGTCAGGAACAGATGGAACAGCTGAATATGGGCCAAAGCGGATATC
TGTGGTAAGCAGTTCCTGCCCCGGCTCAGGGCCAAGAACAGATGGAACAGCTGAATATGGGCCAAACAGGATATCT
GTGGTAAGCAGTTCCTGCCCCGGCTCAGGGCCAAGAACAGATGGTCCCCAGATGCGGTCCAGCCCTCAGCAGTTTC
TAGAGAACCATCAGATGTTTCCAGGGTGCCCCAAGGACCTGAAATGACCCTGTGCCTTATTTGAACTAACCAATCAG
TTCGCTTCTCGCTTCTGTTCGCGCGCTTCTGCTCCCCGAGCTCAATAAAAGAGCCCACAACCCCTCACTCGGGGCGC
CAGTCCTCCGATTGACTGAGTCGCCCGGGTACCCGTGTATCCAATAAACCCTCTTGCAGTTGCATCCGACTTGTGGT
CTCGCTGTTCCTTGGGAGGGTCTCCTCTGAGTGATTGACTACCCGTCAGCGGGGGTCTTTCATTTGGGGGCTCGTCC
GGGATCGGGAGACCCCTGCCCAGGGACCACCGACCCACCACCGGGAGGTAAGCTGGCCAGCAACTTATCTGTGTCT
GTCCGATTGTCTAGTGTCTATGACTGATTTTATGCGCCTGCGTCGGTACTAGTTAGCTAACTAGCTCTGTATCTGGCGG
ACCCGTGGTGGAACTGACGAGTTCGGAACACCCGGCCGCAACCCTGGGAGACGTCCCAGGGATCTCGGGGGCCGT
TTTTGTGGCCCGACCTGAGTCCAAAAATCCCGATCGTTTTGGACTCTTTGGTGCACCCCCCTTAGAGGAGGGATATG
TGGTTCTGGTAGGAGACGAGAACCTAAAACAGTTCCCGCCTCCGTCTGAATTTTTGCTTTCGGTTTGGGACCGAAGC
CGCGCCGCGCGTCTTGTCTGCTGCAGCATCGTTCTGTGTTGTCTCTGTCTGACTGTGTTTCTGTATTTGTCTGAAAAT
ATGGGCCCGGGCCAGACTGTTACCACTCCCTTAAGTTTGACCTTAGGTCACTGGAAAGATGTCGAGCGGATCGCTCA
CAACCAGTCGGTAGATGTCAAGAAGAGACGTTGGGTTACCTTCTGCTCTGCAGAATGGCCAACCTTTAACGTCGGA
TGGCCGCGAGACGGCACCTTTAACCGAGACCTCATCACCCAGGTTAAGATCAAGGTCTTTTCACCTGGCCCGCATG
GACACCCAGACCAGGTCCCCTACATCGTGACCTGGGAAGCCTTGGCTTTTGACCCCCCTCCCTGGGTCAAGCCCTTT
GTACACCCTAAGCCTCCGCCTCCTCTTCCTCCATCCGCCCCGTCTCTCCCCCTTGAACCTCCTCGTTCGACCCCGCCT
CGATCCTCCCTTTATCCAGCCCTCACTCCTTCTCTAGGCGCCCCCATATGGCCATATGAGATCTTATATGGGGCACCCC
CGCCCCTTGTAAACTTCCCTGACCCTGACATGACAAGAGTTACTAACAGCCCCTCTCTCCAAGCTCACTTACAGGCT
CTCTACTTAGTCCAGCACGAAGTCTGGAGACCTCTGGCGGCAGCCTACCAAGAACAACTGGACCGACCGGTGGTAC
CTCACCCTTACCGAGTCGGCGACACAGTGTGGGTCCGCCGACACCAGACTAAGAACCTAGAACCTCGCTGGAAAG
GACCTTACACAGTCCTGCTGACCACCCCCACCGCCCTCAAAGTAGACGGCATCGCAGCTTGGATACACGCCGCCCA
CGTGAAGGCTGCCGACCCCGGGGGTGGACCATCCTCTAGACTGCCGGATCCCAGTGTGGTGGTACGGGAATTCTGC
AGTCGACGGTACCGCGGGCCCGGGATCCACCGGTCGCCACCAATAAAATATCTTTATTTTCATTACATCTGTGTGTTG
GTTTTTTGTGTGAATCGATAGTACTAACATACGCTCTCCATCAAAACAAAACGAAACAAAACAAACTAGCAAAATAG
GCTGTCCCCAGTGCAAGTGCAGGTGCCAGAACATTTCTCTGGCCTAACTGGCCGGTACCTGAGCTCGCTAGCCTCG
AGGATATCAAGATCTGGCCTCGGCGGCCAAGCTTAGACACTAGAGGGTATATAATGGAAGCTCGACTTCCAGCTTGG
CAATCCGGTACTGTTGGTAAAGCCACCATGGTGAGCAAGGGCGAGGAGGATAACATGGCCATCATCAAGGAGTTCA
TGCGCTTCAAGGTGCACATGGAGGGCTCCGTGAACGGCCACGAGTTCGAGATCGAGGGCGAGGGCGAGGGCCGCC
CCTACGAGGGCACCCAGACCGCCAAGCTGAAGGTGACCAAGGGTGGCCCCCTGCCCTTCGCCTGGGACATCCTGTC
CCCTCAGTTCATGTACGGCTCCAAGGCCTACGTGAAGCACCCCGCCGACATCCCCGACTACTTGAAGCTGTCCTTCC
CCGAGGGCTTCAAGTGGGAGCGCGTGATGAACTTCGAGGACGGCGGCGTGGTGACCGTGACCCAGGACTCCTCCC
TGCAGGACGGCGAGTTCATCTACAAGGTGAAGCTGCGCGGCACCAACTTCCCCTCCGACGGCCCCGTAATGCAGAA
GAAGACCATGGGCTGGGAGGCCTCCTCCGAGCGGATGTACCCCGAGGACGGCGCCCTGAAGGGCGAGATCAAGCA
GAGGCTGAAGCTGAAGGACGGCGGCCACTACGACGCTGAGGTCAAGACCACCTACAAGGCCAAGAAGCCCGTGC
AGCTGCCCGGCGCCTACAACGTCAACATCAAGTTGGACATCACCTCCCACAACGAGGACTACACCATCGTGGAACA
GTACGAACGCGCCGAGGGCCGCCACTCCACCGGCGGCATGGACGAGCTGTACAAGTAATAATTCTAGAGTCGGGGC
GGCCGGCCGCTTCGAGCAGACATGATAAGATACATTGATGAGTTTGGACAAACCACAACTAGAATGCAGTGAAAAA
AATGCTTTATTTGTGAAATTTGTGATGCTATTGCTTTATTTGTAACCATTATAAGCTGCAATAAACAAGTTAACAACAA
CAATTGCATTCATTTTATGTTTCAGGTTCAGGGGGAGGTGTGGGAGGTTTTTTAAAGCAAGTAAAACCTCTACAAAT
GTGGTAAGCGGCCGCCAGCACAGTGGTCGACGATAAAATAAAAGATTTATTTAGTCTCCAGAAAAAGGGGGGAAT

GAAAGACCCCACCTGTAGGTTTGGCAAGCTAGCTTAAGTAACGCCATTTTGGAAGGCATGGAAAAATACATAACTG
AGAATAGAGAAGTTCAGATCAAGGTCAGGAACAGATGGAACAGCTGAATATGGGCCAAACAGGATATCTGTGGTAA
GCAGTTCCTGCCCCGGCTCAGGGCCAAGAACAGATGGAACAGCTGAATATGGGCCAAACAGGATATCTGTGGTAAG
CAGTTCCTGCCCCGGCTCAGGGCCAAGAACAGATGGTCCCCAGATGCGGTCCAGCCCTCAGCAGTTTCTAGAGAAC
CATCAGATGTTTCCAGGGTGCCCCAAGGACCTGAAATGACCCTGTGCCTTATTTGAACTAACCAATCAGTTCGCTTC
TCGCTTCTGTTCGCGCGCTTCTGCTCCCCGAGCTCAATAAAAGAGCCCACAACCCCTCACTCGGGGCGCCAGTCCTC
CGATTGACTGAGTCGCCCGGGTACCCGTGTATCCAATAAACCCTCTTGCAGTTGCATCCGACTTGTGGTCTCGCTGTT
CCTTGGGAGGGTCTCCTCTGAGTGATTGACTACCCGTCAGCGGGGGTCTTTCATTCTGCATTAATGAATCGGCCAAC
GCGCGGGGAGAGGCGGTTTGCGTATTGGGCGCTCTTCCGCTTCCTCGCTCACTGACTCGCTGCGCTCGGTCGTTCGG
CTGCGGCGAGCGGTATCAGCTCACTCAAAGGCGGTAATACGGTTATCCACAGAATCAGGGGATAACGCAGGAAAGA
ACATGTGAGCAAAAGGCCAGCAAAAGGCCAGGAACCGTAAAAAGGCCGCGTTGCTGGCGTTTTTCCATAGGCTCC
GCCCCCCTGACGAGCATCACAAAAATCGACGCTCAAGTCAGAGGTGGCGAAACCCGACAGGACTATAAAGATACC
AGGCGTTTCCCCCTGGAAGCTCCCTCGTGCGCTCTCCTGTTCCGACCCTGCCGCTTACCGGATACCTGTCCGCCTTTC
TCCCTTCGGGAAGCGTGGCGCTTTCTCATAGCTCACGCTGTAGGTATCTCAGTTCGGTGTAGGTCGTTCGCTCCAAG
CTGGGCTGTGTGCACGAACCCCCCGTTCAGCCCGACCGCTGCGCCTTATCCGGTAACTATCGTCTTGAGTCCAACCC
GGTAAGACACGACTTATCGCCACTGGCAGCAGCCACTGGTAACAGGATTAGCAGAGCGAGGTATGTAGGCGGTGCT
ACAGAGTTCTTGAAGTGGTGGCCTAACTACGGCTACACTAGAAGGACAGTATTTGGTATCTGCGCTCTGCTGAAGCC
AGTTACCTTCGGAAAAAGAGTTGGTAGCTCTTGATCCGGCAAACAAACCACCGCTGGTAGCGGTGGTTTTTTTGTTT
GCAAGCAGCAGATTACGCGCAGAAAAAAAGGATCTCAAGAAGATCCTTTGATCTTTTCTACGGGGTCTGACGCTCA
GTGGAACGAAAACTCACGTTAAGGGATTTTGGTCATGAGATTATCAAAAAGGATCTTCACCTAGATCCTTTTGCGGC
CGGCCGCAAATCAATCTAAAGTATATATGAGTAAACTTGGTCTGACAGTTACCAATGCTTAATCAGTGAGGCACCTAT
CTCAGCGATCTGTCTATTTCGTTCATCCATAGTTGCCTGACTCCCCGTCGTGTAGATAACTACGATACGGGAGGGCTT
ACCATCTGGCCCCAGTGCTGCAATGATACCGCGAGACCCACGCTCACCGGCTCCAGATTTATCAGCAATAAACCAGC
CAGCCGGAAGGGCCGAGCGCAGAAGTGGTCCTGCAACTTTATCCGCCTCCATCCAGTCTATTAATTGTTGCCGGGAA
GCTAGAGTAAGTAGTTCGCCAGTTAATAGTTTGCGCAACGTTGTTGCCATTGCTACAGGCATCGTGGTGTCACGCTC
GTCGTTTGGTATGGCTTCATTCAGCTCCGGTTCCCAACGATCAAGGCGAGTTACATGATCCCCCATGTTGTGCAAAA
AAGCGGTTAGCTCCTTCGGTCCTCCGATCGTTGTCAGAAGTAAGTTGGCCGCAGTGTTATCACTCATGGTTATGGCA
GCACTGCATAATTCTCTTACTGTCATGCCATCCGTAAGATGCTTTTCTGTGACTGGTGAGTACTCAACCAAGTCATTC
TGAGAATAGTGTATGCGGCGACCGAGTTGCTCTTGCCCGGCGTCAACACGGGATAATACCGCGCCACATAGCAGAA
CTTTAAAAGTGCTCATCATTGGAAAACGTTCTTCGGGGCGAAAACTCTCAAGGATCTTACCGCTGTTGAGATCCAGT
TCGATGTAACCCACTCGTGCACCCAACTGATCTTCAGCATCTTTTACTTTCACCAGCGTTTCTGGGTGAGCAAAAAC
AGGAAGGCAAAATGCCGCAAAAAAGGGAATAAGGGCGACACGGAAATGTTGAATACTCATACTCTTCCTTTTTCAA
TATTATTGAAGCATTTATCAGGGTTATTGTCTCATGAGCGGATACATATTTGAATGTATTTAGAAAAATAAACAAATAG
GGGTTCCGCGCACATTTC

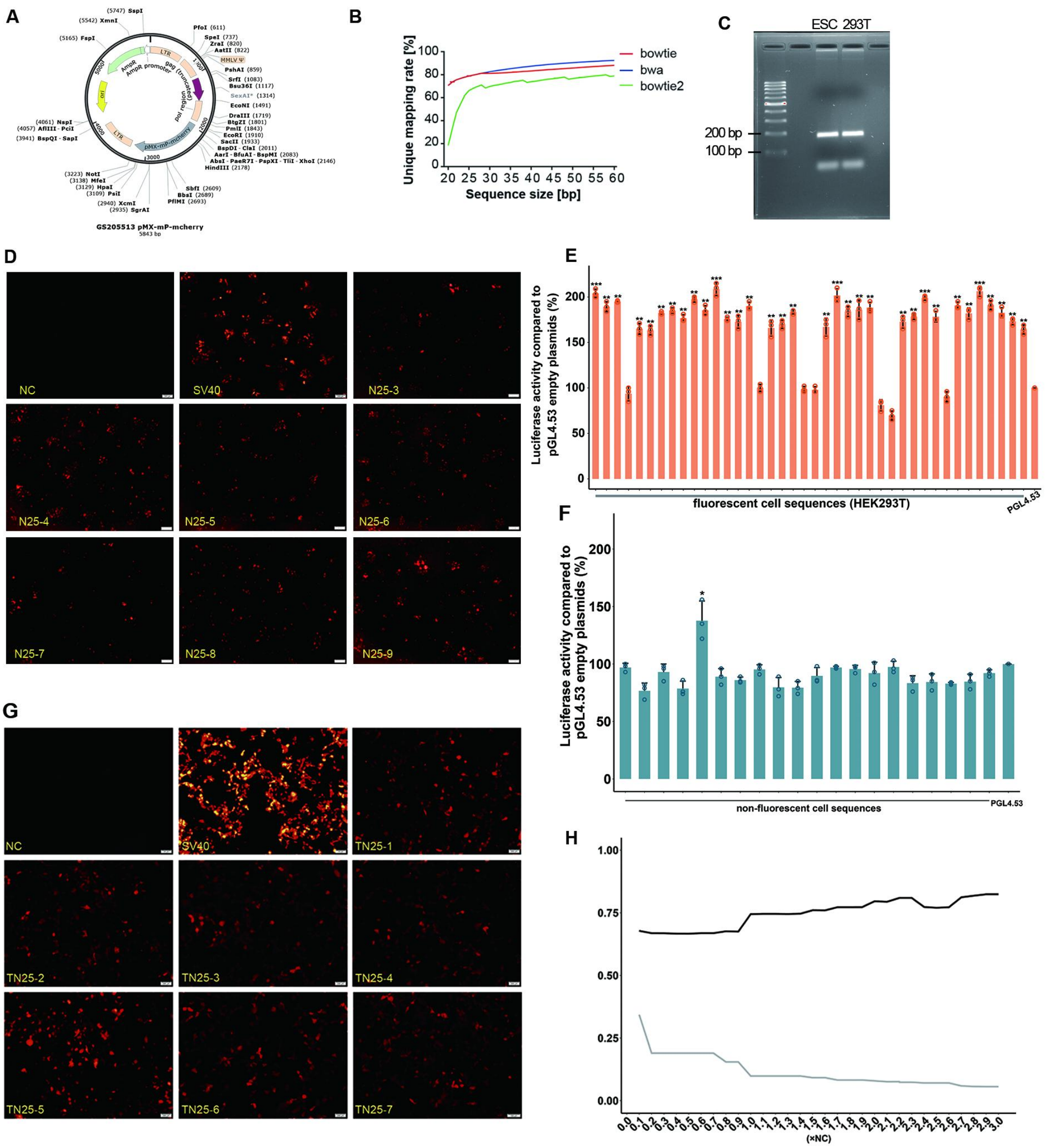


**Fig. S1. Supporting materials related to MAE-seq development.**

(A) Plasmid map of pMX-mp-mcherry reporter vector.

(B) Test for sequence length and alignment software. DNA sequences of 20-60 base pairs length are randomly extracted from the hg19 genome. Statistics of unique mapping rates are conducted with bowtie, bowtie2 and bwa software, respectively.

(C) Amplification of sequencing library of positive cells. ESC, represents positive mESCs; 293T, represents positive HEK 293T cells.

(D) Fluorescence expression in mESCs Validation of N25-3 to N25-9 supplemented to figure 1C.

(E) 40 MAE-seq loci were randomly selected in HEK293T for activity validation using the luciferase system. empty pGL4.53 plasmid was used as the control for baseline luciferase activity. The y axis represents the percentage of luciferase activity compared to pGL4.53 empty plasmids in the respective cells (n = 3 biological independent samples; bars show mean value ± s.e.m.; ns: non-significant, **:p<0.01, calculated using t-test).

(F) 20 sequences enriched in non-fluorescent cells were validated for enhancer activity by the luciferase system. empty pGL4.53 plasmid was used as the control for baseline luciferase activity. The y axis represents the percentage of luciferase activity compared to pGL4.53 empty plasmids in the respective cells (n = 3 biological independent samples; bars show mean value ± s.e.m.; ns: non-significant, *:p<0.05, calculated using t-test).

(G) MAE-seq sites were randomly selected in HEK293T cell for fluorescence expression verification. These sites were constructed upstream of the mini promoter of mCherry and transfected into HEK293T cells, and the fluorescence expression was observed 48 hours later. NC is negative control.

(H) The black curve is the product of normalized overlap signal/non-overlap signal(y) and normalized residual rate of overlap signal(y). And the formula is as follow:

$$F(x,y) = \frac{x - 1.75}{1.9 - 1.75} \times 2^y F(x,y) = \frac{x - 1.75}{1.9 - 1.75} \times 2^y$$

The grey curve is residual rate of overlap signal.

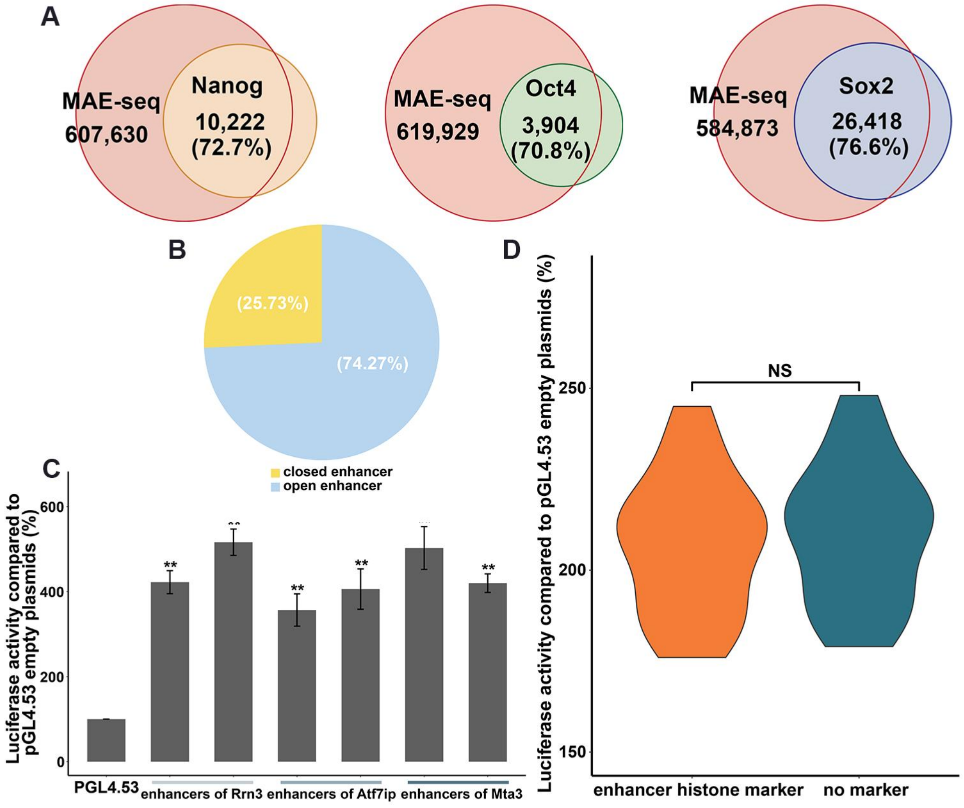


**Fig. S2. Epigenetic characteristics and chromatin accessibility of MAE-seq loci.**

(A) The percentage of different ES-specific TF covered by MAE-seq sites.

(B) The percentage of enhancers involved in closed chromatin and open chromatin.

(C) Activity of enhancers in heterochromatin regions interacting with housekeeping genes. empty pGL4.53 plasmid was used as the control for baseline luciferase activity. The y axis represents the percentage of luciferase activity compared to pGL4.53 empty plasmids in the respective cells (n = 3 biological independent samples; bars show mean value ± s.e.m.; ns: non-significant, **:$p<0.01$, calculated using t-test).

(D)The relative Luciferase activity distribution of 25bp MAE-seq enhancers with enhancer histone markers and 25bp MAE-seq enhancers with no enhancer histone

markers. T-test was used for difference analysis.

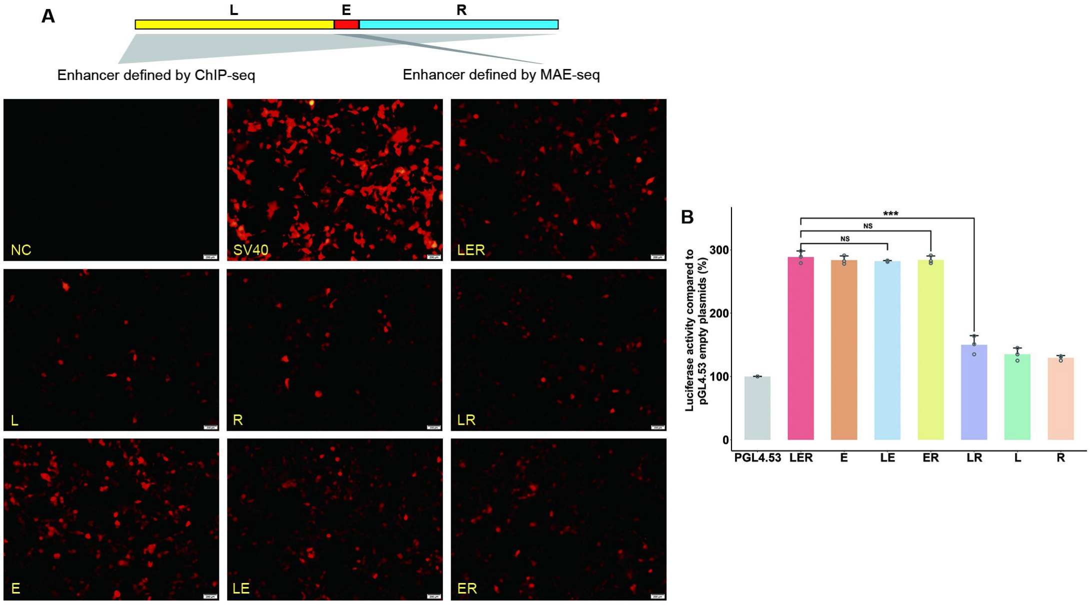


**Fig.S3. Activity validation of MAE-seq enhancers with or without left or right flanking sequences in HEK 293T cell.**

(A) Enhancer activity of different fragments in "LER" experiments after transfection of mESCs. Different fragments were constructed into the reporter vector with mCher gene and transfected into cells, and the fluorescence expression was observed after 48 hours. NC, empty reporter vector; SV40, SV40 enhancer; L, left flanking sequence; R, right flanking sequence; E, 25bp enhancer.

(B) Differential analysis of enhancer activity of different fragments in "LER" experiment by luciferase. L, left flanking sequence; R, right flanking sequence; E, 25bp enhancer. empty pGL4.53 plasmid was used as the control for baseline luciferase activity. The y axis represents the percentage of luciferase activity compared to pGL4.53 empty plasmids in the respective cells (n = 3 biological independent samples; bars show mean value ± s.e.m.; ns: non-significant, ***:

p<0.001, calculated using t-test).

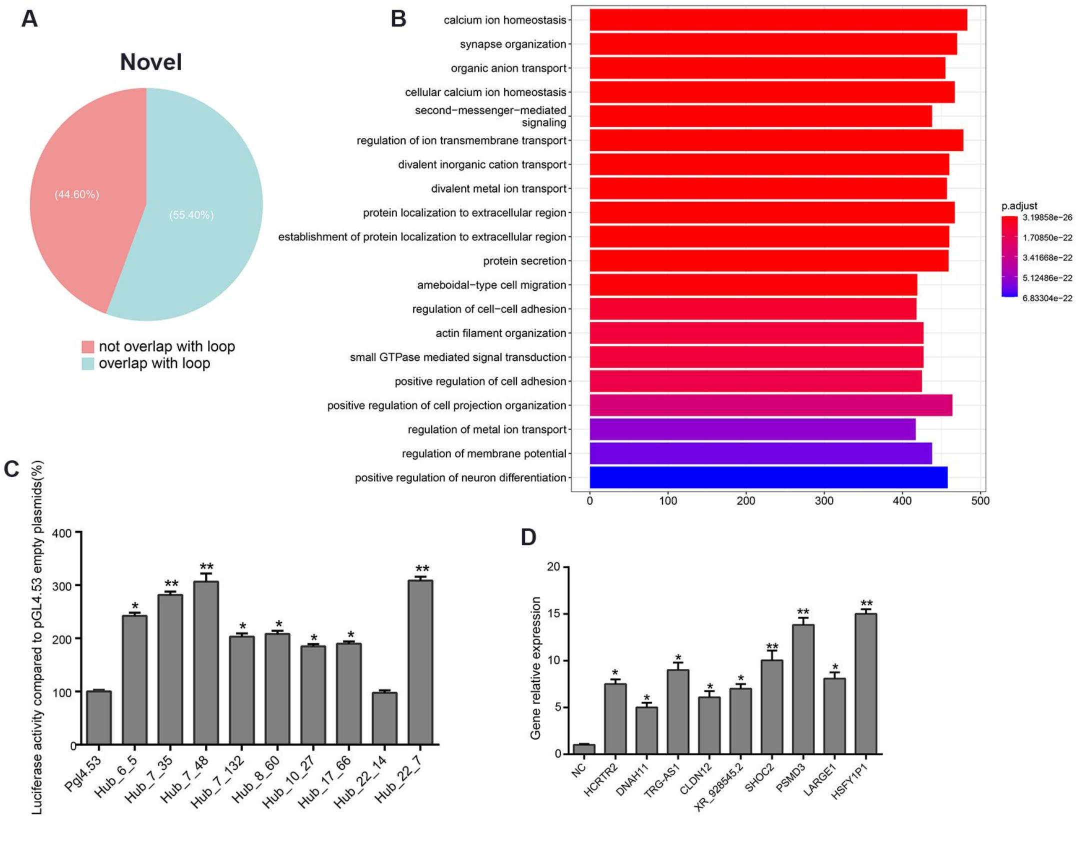


**Fig.S4. Participate in the ratio of chromatin loop novel enhancers and the GO analysis of known enhancers.**

(A) The percentage of novel enhancer overlapped with chromatin loop anchor.

(B) GO enrichment analysis of nearest genes related to known enhancers.

(C) Double luciferase verification results of 9 sites in Hub region. The y axis represents the percentage of luciferase activity compared to pGL4.53 empty plasmids in the respective cells (n = 3 biological independent samples; bars show mean value ± s.e.m.; ns: non-significant, *:p<0.05, **: p<0.01, calculated using t-test).

(D) After the dCas9-VP64 system activated the 9 regions in ( C ), QPCR was used to detect the expression level of the interacting target genes.(*: p<0.05; **: p<0.01, calculated using t-test )

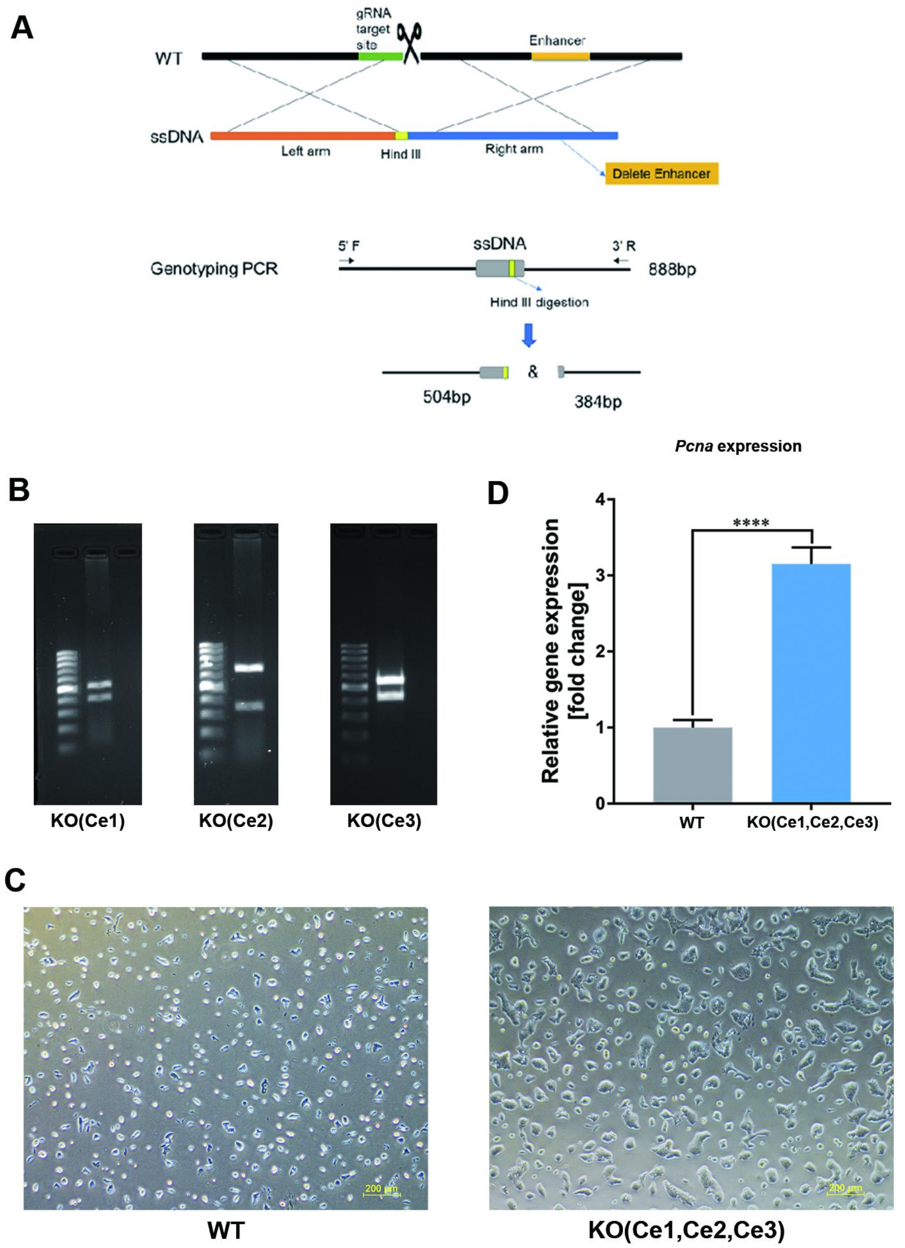


**Fig.S5. Ce1 to Ce3 knockout results supplement to figure 5.**

(A) Experimental procedure of enhancer knockout based on CRSPR/Cas9 and ssDNA. Three MAE-seq enhancers (Ce1, Ce2 and Ce3) targeted to Cdh1 are verified in Hi-C data. This procedure takes Ce1 as example. WT, wild type locus; ssDNA,

donor single strand DNA without enhancer; Hind III, introduced restriction sites in ssDNA; 5’F, 3’R, forward and reverse primer for genotyping PCR.

(B) Genotyping PCR of Ce1 to Ce3 knockout in mESCs.

(C) Proliferation phenotype of triple knockout cell.

(D) Expression of *Pcna* gene of triple knockout cell. ****: $p < 0.0001$, calculated using t-test.

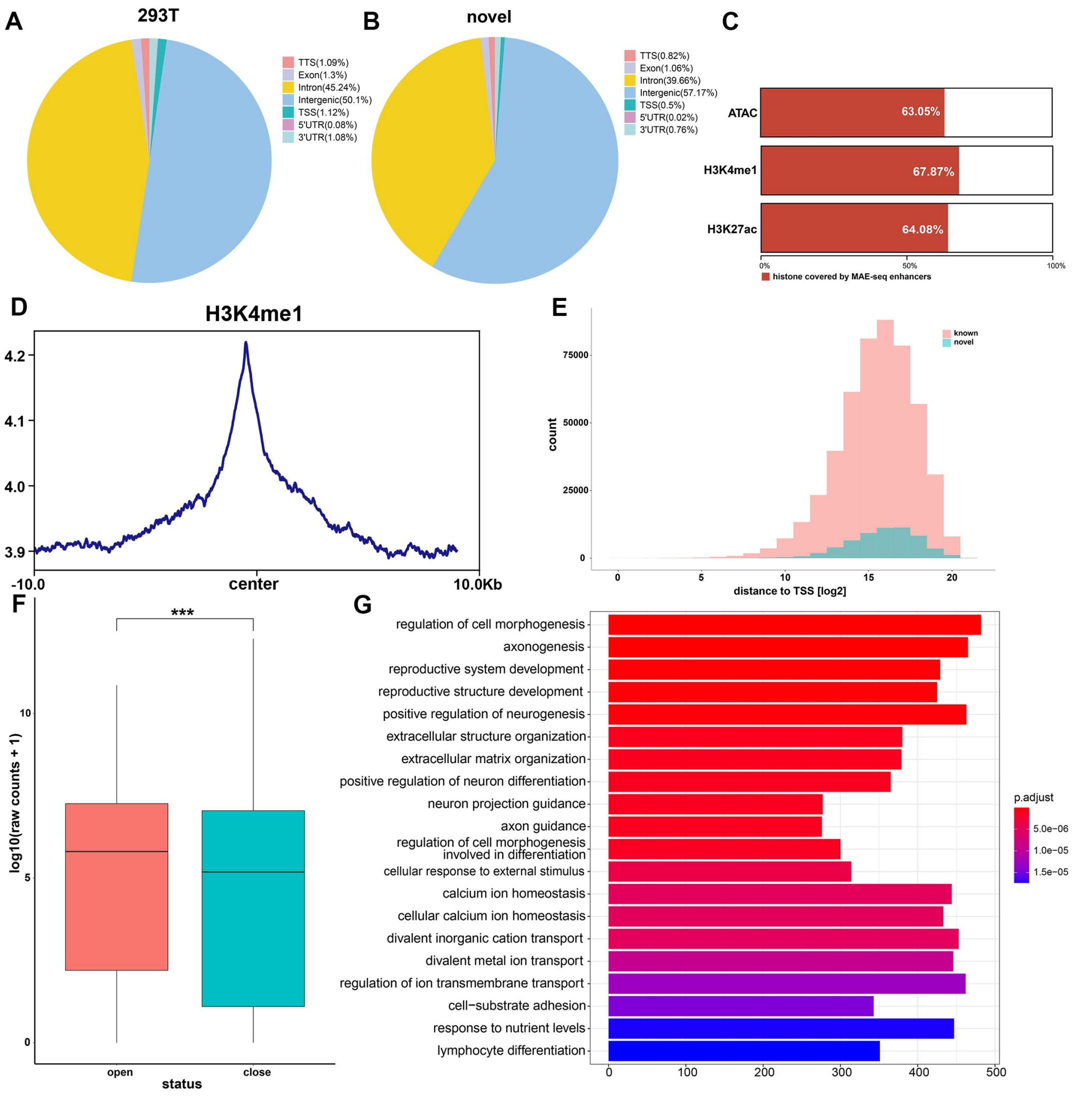


**Fig. S6. Epigenetic characterization and taxonomic analysis of HEK 293T.**

(A) Genomic distribution of HEK 293T MAE-seq enhancers.

(B) Genomic distribution of HEK 293T MAE-seq novel enhancers.

(C) The percentage of different enhancer epigenetic markers covered by HEK 293T MAE-seq sites.

(D) The enrichment of H3K4me1 marks around the HEK 293T MAE-seq loci.

(E) Statistics of distance to TSS of HEK 293T novel enhancers. The two types of enhancers did not differ significantly from the site of TSS. Red column represents known mouse enhancers; the blue column represents novel enhancers.

(F) Expression of nearest genes related to HEK 293T MAE-seq enhancers which are in open and closed chromatin regions. Open, represent ATAC; closed, represent none of ATAC. ***, $p<0.001$.

(G) GO enrichment analysis of nearest genes related to HEK 293T enhancers.

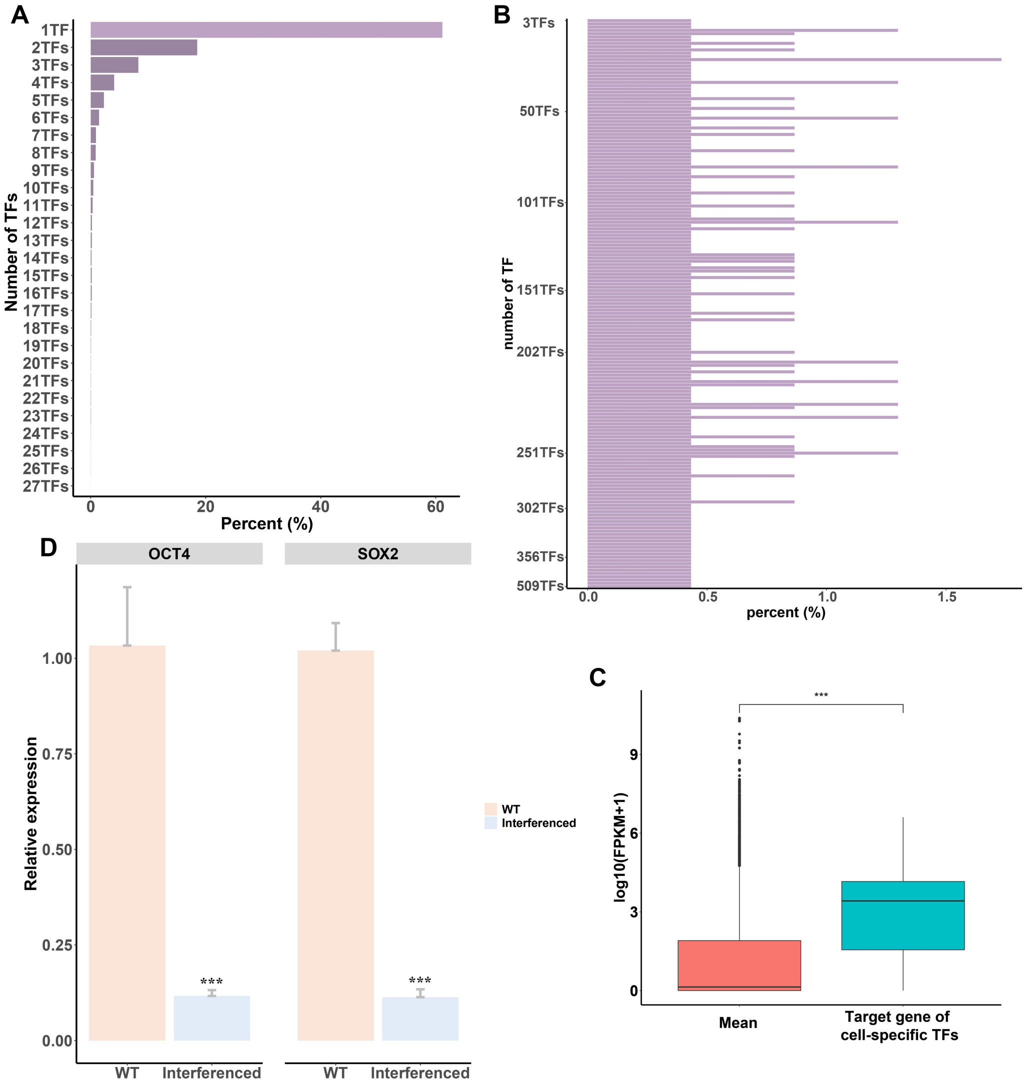


**Fig.S7 The number of TFs bound to long sequences and the expression of target cofactors after interference with cells.**

(A) The percentage statistics of the number of combined TFs on the MAE-seq enhancer sequence in mESCs.

(B) The percentage statistics of the number of combined TFs on the Super-Enhancers enhancer sequence in mESCs.

(C) The average level of target genes corresponding to single cell-specific TF enhancers was compared with that of genomic genes.

(D) QPCR was used to detect the change of target gene *OCT4* and *CDH1* expression

after Cas13d interference. WT, wildtype cell ; interferenced, Cas13d RNAi cell, ***$p < 0.001$.
